# Enteric Neuronal Substrates Underlying Spontaneous and Evoked Neurogenic Contractions in Mouse Colon

**DOI:** 10.1016/j.jcmgh.2025.101462

**Published:** 2025-01-13

**Authors:** Sushmita Debnath, Dante J. Heredia, Nicole M. Procacci, Camila Fedi, Emer P. Ni Bhraonain, Caroline A. Cobine, Thomas W. Gould

**Affiliations:** Department of Physiology and Cell Biology, University of Nevada Reno School of Medicine, Reno, Nevada

**Keywords:** Calcium Imaging, Cholinergic, Colonic Motility, Nitrergic

## Abstract

**Background & Aims:**

Gastrointestinal motility persists when peripheral cholinergic signaling is blocked genetically or pharmacologically, and a recent study suggests nitric oxide drives propagating neurogenic contractions.

**Methods:**

To determine the neuronal substrates that underlie these contractions, we measured contractile-associated movements together with calcium responses of cholinergic or nitrergic myenteric neurons in unparalyzed *ex vivo* preparations of whole mouse colon. We chose to look at these 2 subpopulations because they encompass nearly all myenteric neurons.

**Results:**

Many, but not all, cholinergic neurons of the middle colon exhibited contractile-associated calcium responses with distinct features. By contrast, a large population of nitrergic neurons of the middle colon shut their activity off just before contraction onset, whereas another population of nitrergic neurons initiated a response just after contraction onset. When contractions were evoked by a variety of stimuli to the proximal and distal colon, the same neuronal subtypes exhibited the same activity patterns during the contraction. However, stimulation of proximal colon produced a transient, stimulation-locked response before the ensuing contraction in a subpopulation of cholinergic neurons and in nearly all nitrergic neurons, suggesting that distinct neuronal activity patterns underlie specific stimuli. Finally, although blockade of nitric oxide failed to arrest the generation or propagation of neurogenic contractions, chemogenetic elimination of nitrergic activity impaired their propagation to middle and distal colon.

**Conclusions:**

Genetic approaches were used to study the activity patterns of enteric neurons underlying spontaneous and evoked neurogenic contractions in unparalyzed colon. These approaches can be combined with a variety of other approaches to identify the neuronal subtypes and subclasses that coordinate colonic motility.

**Summary**

Using cell-specific tools to image myenteric neurons of mouse colon, we find that distinct subtypes are active during spontaneous neurogenic contractions. The same neurons were active during evoked contractions, suggesting the existence of a common contractile neuronal substrate.

Activity patterns intrinsic to the enteric nervous system (ENS) that generate gastrointestinal motility have been extensively investigated in many organisms and regions of gut. Both neurogenic substrates residing in the myenteric plexus and myogenic substrates originating within interstitial cells of Cajal (ICCs) contribute to specific motor patterns. In the colon, spontaneous motor patterns include low-frequency, large-amplitude, long-duration neurogenic contractions that propagate anterogradely along the colon, separated by periods of neurogenic tonic inhibition,^1^ and high-frequency, small-amplitude, short-duration myogenic phasic contractions.^2^^,^^3^ Smooth muscle cells and ICCs express receptors for neurotransmitters, providing a link for the coordination of neurogenic and myogenic motor patterns.^4^ The functional and clinical importance of neurogenic motor patterns is underscored by the severe defects in motility, such as constipation and bowel obstruction, observed in conditions characterized by the developmental loss of colonic myenteric neurons, such as Hirschsprung’s disease.^5^

Although the molecular and cellular mechanisms underlying myogenic contractions in the gut have been extensively characterized,^6^ the precise nature of the substrates underlying neurogenic contractions, termed migrating motor complexes (MMCs),^7^ remains elusive. Electrophysiologic recordings of smooth muscle cells in paralyzed preparations of whole mouse colon showed that the colonic MMC (CMMC) is characterized by rapid oscillations superimposed on a slow depolarization,^8^ sometimes but not always preceded by a precomplex hyperpolarization. The slow depolarization appears to be mediated by the cessation of the release of inhibitory neurotransmitters, such as nitric oxide (NO), during the period of tonic inhibition in between CMMC.^9^^,^^10^ In contrast, rapid oscillations are produced by activation of cholinergic excitatory motor neurons and the transduction of this activity by muscarinic acetylcholine (ACh) receptors in the ICCs and smooth muscle, as they are blocked by atropine.^8^^,^^9^^,^^11^

Despite the effects of muscarinic blockade on electrical activity, CMMCs are reduced but not abolished in atropine,^12^ suggesting that non-muscarinic mechanisms may contribute to this motor pattern. One possible molecule(s) mediating atropine-resistant contractions are tachykinins that are co-released with ACh by excitatory motor neurons.^13^, ^14^, ^15^ However, the tools used to disrupt these pathways peripherally at the neuroeffector junction may also affect signaling centrally within the myenteric ganglia themselves, as these neurons express multiple neurokinin receptor subtypes.^16^ Alternatively, the disinhibition of smooth muscle, caused by the cessation of inhibitory neurotransmitter release, may contribute to CMMCs. However, CMMC frequency is increased in the absence of the gene encoding the NO biosynthetic enzyme nitric oxide synthase 1 (Nos1), or in the presence of various NO antagonists,^12^^,^^14^^,^^15^ suggesting that blockade of peripheral NO release itself is unlikely to generate CMMCs (but see Koh et al^17^). A recent imaging study demonstrated that all myenteric neurons, both cholinergic and nitrergic, are active at a frequency of 2 Hz during CMMC.^18^

To address these questions, we developed an imaging preparation that allowed us to simultaneously track the movements of neurogenic contractions and the cholinergic and nitrergic neuronal substrates underlying them. We observed distinct subtypes of activity within each of these 2 neuronal populations during spontaneous neurogenic contractions (spont NgCs). The same subtypes were also activated by mucosal stimulation and stretch of proximal or distal colon, electrical field stimulation (EFS) of the distal colon, and pelvic nerve stimulation (PNS). In addition to this common contractile neuronal substrate, we observed distinct patterns of activity before contractions that were elicited by different stimuli. In order to determine whether NO or nitrergic activity is required for neurogenic contractions, we analyzed propagation of them by tension recording in wild-type mice treated with NO antagonists or in mice whose nitrergic neurons were chemogenetically inactivated. In contrast to the blockade of NO itself, which failed to disrupt these contractions, silencing their activity impaired the propagation of them to the middle and distal colon. Together, these genetic approaches represent a powerful approach toward the elucidation of the neurogenic substrates underlying gastrointestinal motility.

## Results

### Characterizing Spontaneous and Evoked Colonic Contractions in CAGGS-GCaMP3 Mice

**Figure 1:**
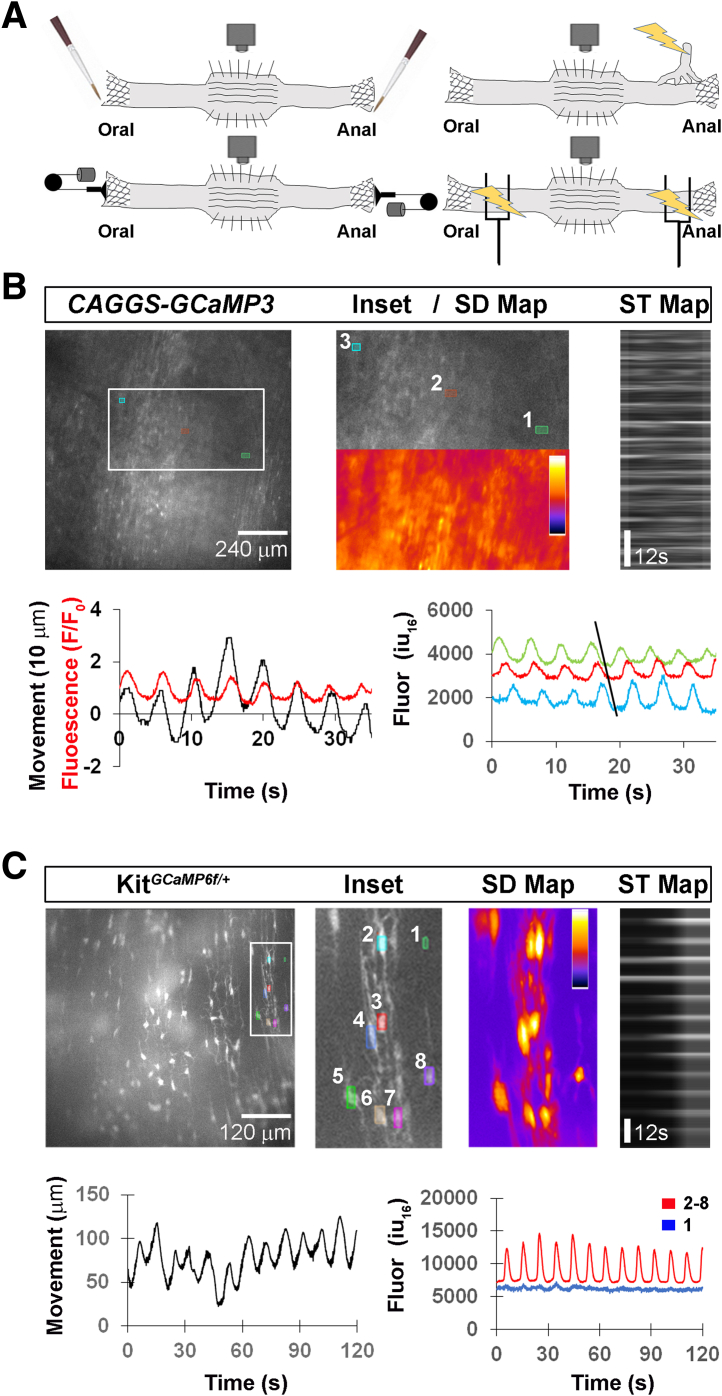
**Spontaneous myogenic movements of the middle colon coincide with Ca**^**2+**^**responses in CM and ICCs.** (*A*) Schematic of colon preparation used for imaging myogenic and neurogenic contractile movements as well as cell-specific Ca^2+^ responses. In all preparations, the proximal or oral colon is to the left of the middle colon being imaged, and the distal or anal colon is to the right. Middle colon is split open and pinned with mucosa-side down. Spontaneous movements are imaged, as are those evoked by oral or anal mucosal stimulation with a brush (*top left*), by oral or anal longitudinal stretch (*bottom left*), by stimulation of the pelvic nerve with suction electrode (*top right*), or by transmural EFS of the regions near the proximal or distal ends of the colon (*bottom right*). (*B*) Cross-section of middle colon from *CAGGS-GCaMP3* mice (*left top*), imaged at 10×, and inset box showing 3 ROIs, and corresponding area showing SD map of fluorescence intensity (*middle top*), and ST map of fluorescence intensity from ROI 1. Fire LUT scale values (iu_16_) for SD map: white = 21,000; blue = 3000). *White lines* in ST map depict rhythmic nature of Ca^2+^ responses of CM. Movement (*black*) and average fluorescence responses of CM ROIs 1-3 (*gray*) show temporal coincidence (*bottom left*). Individual transients from ROI 1-3 show a retrogradely travelling contraction (noted by diagonal line in bottom right graph); transients were moved up or down y-axis to illustrate this propagation, so starting and ending values are not accurate, but trough-to-peak amplitudes and frequencies are. (*C*) Cross-section of middle colon from *Kit-GCaMP6f* mice (*left top*), imaged at 20×, and inset box showing 8 ROIs (*middle left top*), and corresponding area showing SD map (*middle right top*), and ST map of fluorescence intensity from ROI 2. *White lines* in ST map depict rhythmic nature of ICC Ca^2+^ responses of CM. Movement (*black, left bottom*) and fluorescence response of ICC (average of ROIs 2–8; *red*) also exhibit temporal coincidence (ROI 1 = background; *blue*). ICC transient frequency, 12 ± 3.4/min; amplitude = 4163 ± 2370 iu_16_; c = 35; n = 2 M, 2 F. Fire LUT scale values (iu_16_) for SD map: white = 14,500; blue = 5300).

We first examined the middle colon of *CAGGS-GCaMP3* mice, which drive expression of GCaMP3 in most cell types (see Methods).^19^ We recorded 2-minute videos and measured calcium (Ca^2+^) responses as changes in fluorescence of regions of interest (ROIs) and movement as changes in position or displacement of selected ROIs. One class of movement occurred with a magnitude of 35.6 ± 13.8 μm (n = 3 males [M], 2 females [F]) and a frequency of 10.3 ± 2.2/min (n = 9 M, 10 F) (Figure 1*B*). These movements occurred in both the anterograde and retrograde direction and were not abolished by the addition of tetrodotoxin (TTX; 1 μM), which blocks most inputs to the colon (data not shown; n = 2 M, 3 F). In addition to measuring movement, we also imaged Ca^2+^ responses in circular smooth muscle cells (CM), which mediate circumferential stretch. CM Ca^2+^ responses (amplitude = 2055 ± 825 iu_16_; c = 32; n = 3 M, 2 F) occurred with the same frequency as that of the movements (Figure 1*B*). We also observed in *CAGGS-GCaMP3* mice a second population of cells whose Ca^2+^ responses occurred contemporaneously as those in CM and as the movements. To determine if these reflected ICC, we imaged Ca^2+^ responses in the colons of mice expressing GCaMP6f in the Kit locus and therefore in ICC. Similar to CM activity, ICC Ca^2+^ responses in *Kit-GCaMP6f* mice produced responses that were of similar frequency as the movements (Figure 1*C*). Together, these data support the idea that this first class of movements are myogenic phasic contractions driven by ICC along the submucosal surface of the circular muscle.^20^, ^21^, ^22^

**Figure 2:**
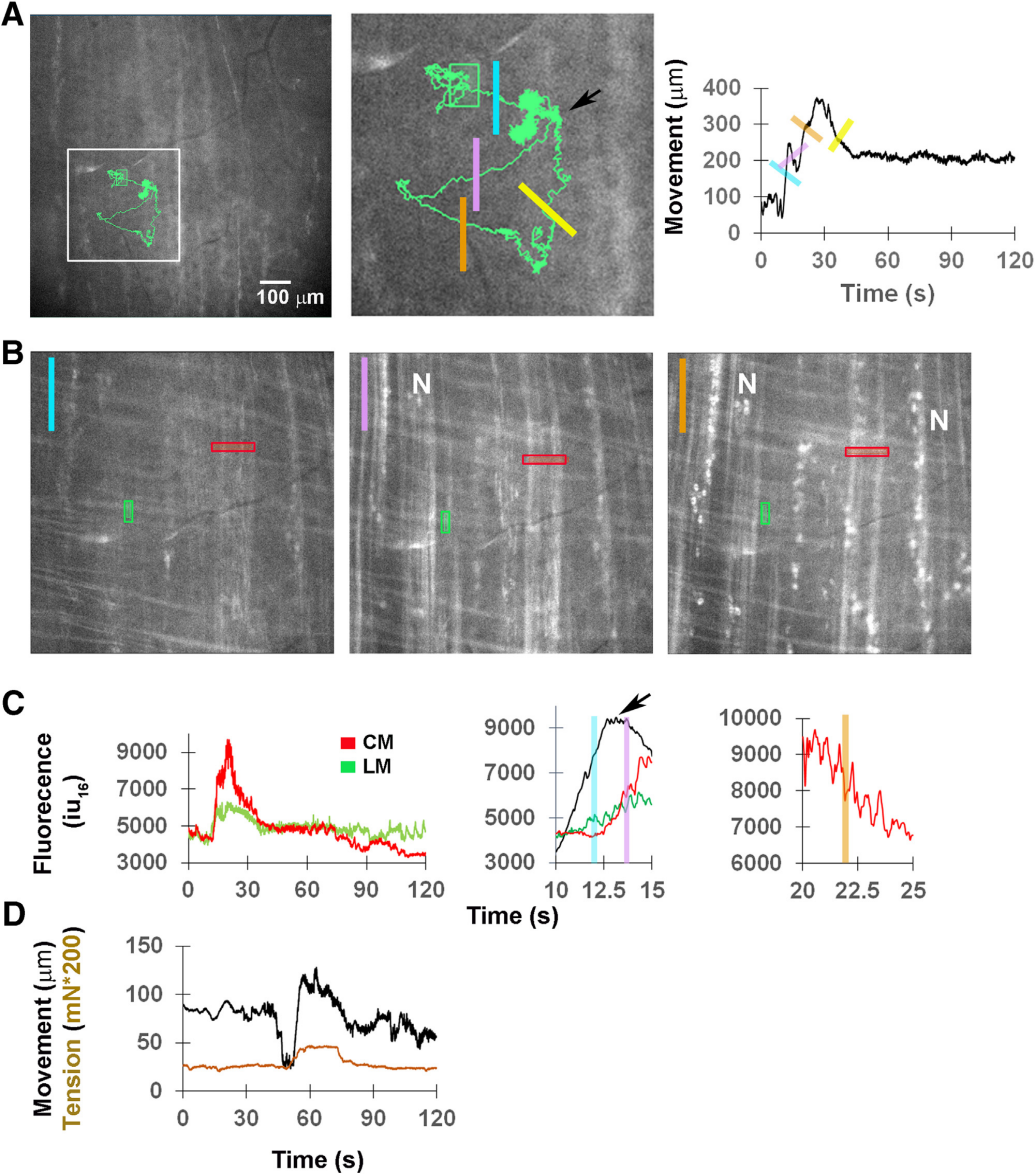
**Spontaneous neurogenic movements of the middle colon are associated with specific temporal patterns of LM and CM Ca**^**2+**^**responses.** (*A*) Image of middle colon from *CAGGS-GCaMP3* mouse with displacement trajectory (*green*) of an ROI used to generate movement track (*graphed in right panel*) as well as motion-stabilized video used for measuring CM and LM Ca^2+^ responses during an Spont NgC (*left panel and inset in middle panel*). Movement duration = 29.4 ± 5.6 seconds; magnitude = 308 ± 92 μm; frequency = 1 per 5.0 ± 1.6 minutes; n = 3 M, 2 F). These contractions were only slightly less frequent than those recorded from the middle whole colon by force transducers during CMMC (every 4.5 ± 0.21 minutes)^12^ and were abolished by the addition of TTX (0 vs 6.3 ± 2.3 large contractions; c = 15; n = 3 M, 3 F), consistent with the neurogenic origin of them. A short time after the first movement stopped (1.75 ± 0.74 seconds; c = 11; n = 3 M, 2 F), the response in LM cells continued, and a second movement initiated, together with Ca^2+^ responses in CM cells that were larger in amplitude than those during phasic contractions (amplitude, 10,998 ± 5526 iu_16_; duration, 21.9 ± 2.9 seconds; c = 41; n = 3 M, 2 F). Subsequently, the movement gradually returned the tissue toward its original position over a variable length of time. Colored lines in middle and right panels indicate sequence of movement: *blue* = first movement to right/distal; *purple* = local contractile movement back left, which continues to the right (orange); *yellow* = relaxation. Note relaxation does not always completely restore original position. *Arrow* indicates end of rightward movement. (*B*) Snapshots of motion-stabilized video taken during rightward movement (*blue; left panel*) and during local contractile movement (*purple, middle panel and orange, right panel*). Note the increase of Ca^2+^ in LM (*green* ROI) during the initial rightward movement (*left panel*), compared with image taken before the movement (*A*, *left panel*). In contrast, CM Ca^2+^ responses (*red* ROI) do not begin until local contractile movement initiates to the left (*middle panel*). Note that myenteric neurons (N) begin their response at the onset of the local contractile movement as well. LM and CM, as well as neurons, maintain the response throughout the local contractile movement (*right panel*). (*C*) Graphs of LM and CM Ca^2+^ responses (*green and red*, respectively), together with movement trace (*black*), during the entire spontaneous contraction (*left*), during its onset (*middle*), or during the local contractile response (*right*). Movement values in middle graph were multiplied by 30 to facilitate comparison to Ca^2+^ values. In the middle graph, note that the initial movement to the right (*blue*) leads the LM Ca^2+^ response, which occurs before the end of this movement (*arrow*), and that CM Ca^2+^ responses do not begin until the onset of the local contractile movement (*purple*). In the right graph, note the individual peaks of the CM Ca^2+^ responses during the second phase of the contractile movement (*orange*). (*D*) Temporal comparison between movement trace (*black*) and tension trace (*brown*) during a spontaneous neurogenic contraction. The magnitude of the movement trace is lower than normal (eg, right panel in *A*) because a small row of pins was placed on the side connected to the force transducer to facilitate movement recording. Note that the second upward deflection, and not the first downward deflection, of the movement is associated with the onset of tension.

In some videos, we observed a second class of contractions whose movements were longer, larger, and less frequent. These contractions sometimes initiated with a small movement, whose onset was closely associated with the onset of a Ca^2+^ response in longitudinal muscle (LM) cells (rightward movement with blue stripe; Figure 2*A–C*; Supplementary Video 1). A second movement then occurred that included a larger movement followed by a period of limited or small movements (bottom right corner of movement track; Figure 2*A*). Collectively, these components of the second movement represent the local contraction, as they were always accompanied by CM Ca^2+^ responses. A final movement lasted longer than the CM response and returned the ROI close to the initial location and thus reflected the relaxation of the contraction (Figure 2*A–C*). To confirm that optically captured periodic movements reflected contractile events, we measured tension with a single force transducer attached to one side of middle colon during them. An initial movement often preceded a second contractile movement, the onset of which coincided with the onset of tension (Figure 2*D*). Together, these studies demonstrate that spont NgCs of the middle colon, presumably a portion of the anterogradely propagating CMMCs that originate in the proximal colon, can be observed in an imaging preparation of the intact mouse colon.

### Contractile and Cholinergic Responses During Tonic Inhibition and Spontaneous Contractions

**Figure 3:**
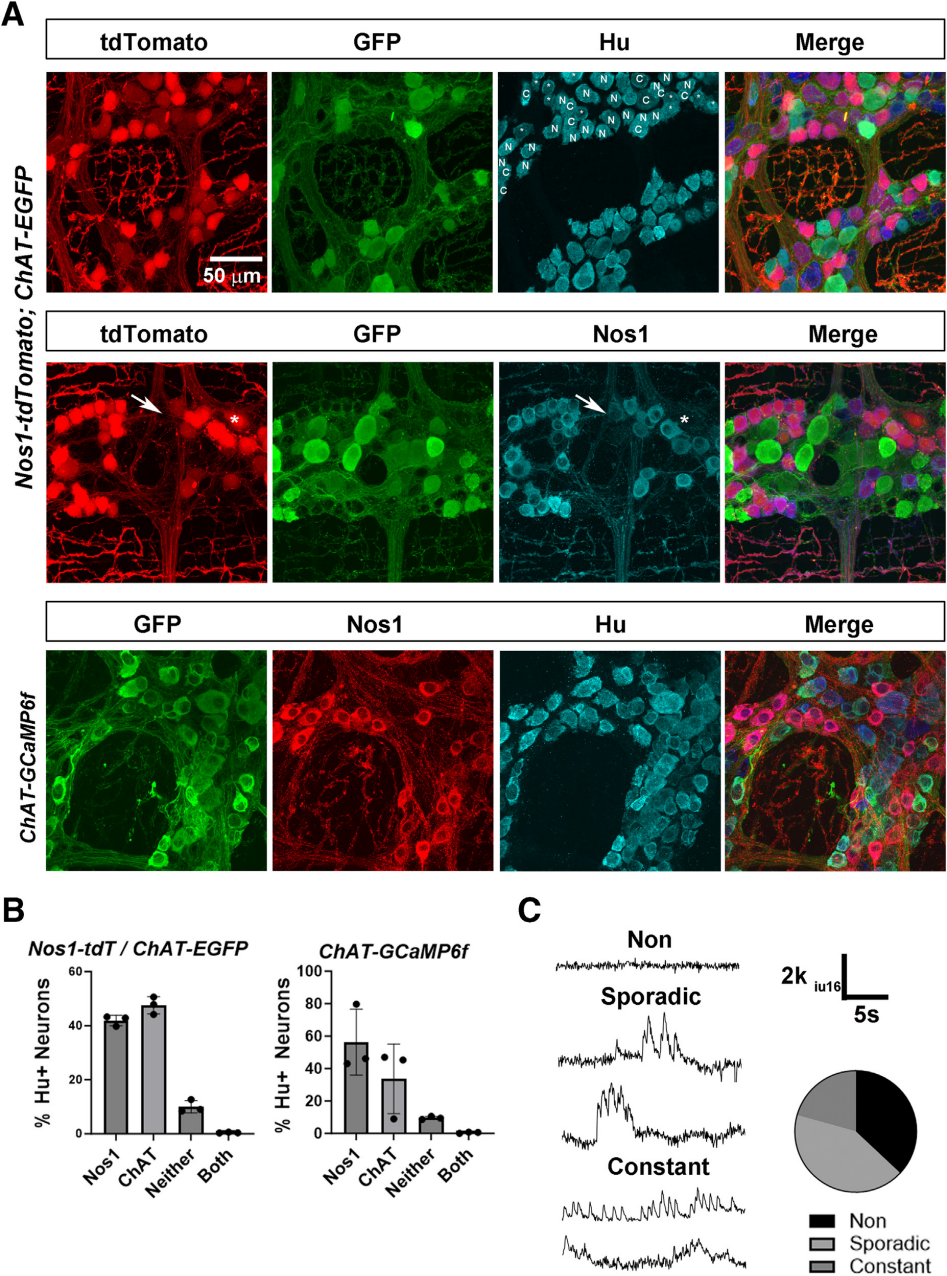
**Genetic labeling of cholinergic and nitrergic myenteric neurons and cholinergic spontaneous activity.** (*A*) Images of myenteric ganglia from *Nos1-tdTomato, ChAT-EGFP* mice. *Top row*, epifluorescence shows nitrergic neurons genetically labeled with the fluorescent reporter tdTomato, cholinergic neurons genetically labeled with EGFP and enhanced with GFP immunofluorescence, and all neurons labeled with Hu immunofluorescence. In the Hu panel, cholinergic or GFP-immunoreactive neurons are labeled C, nitrergic or tdTomato-epifluorescent neurons are labeled N, and cells negative for both tdTomato and GFP are labeled with *asterisks*. No neurons were observed in this image that expressed both fluorescent markers. *Bottom row*, nitrergic and cholinergic neurons labeled as above, and nitrergic neurons also labeled with Nos1 immunofluorescence. Note the occasional cell expressing tdTomato without Nos1 immunoreactivity (*asterisk*), as well as the occasional cell with Nos1 immunoreactivity not expressing tdTomato (*arrow*). Bottom row, mages of myenteric ganglia from *ChAT-GCaMP6f* mice. Cholinergic neurons genetically labeled with GCaMP6f and enhanced with GFP immunofluorescence, nitrergic neurons labeled with Nos1 immunofluorescence, and all neurons labeled with Hu immunofluorescence. (*B*) Quantification of nitrergic and cholinergic neurons, as a percentage of Hu+ neurons, using genetic tools (42 ± 1.9 vs 47.6 ± 3.3 vs 10 ± 2.3 vs 0.4 ± 0.2%; Nos1+ (by tdTomato), ChAT+ (by GFP), double-negative, double-positive neurons in *Nos1-tdTomato, ChAT-EGFP* mice; 44.4 ± 1.6 vs 47.4 ± 2.3 vs 7.9 ± 1.9 vs 0.4 ± 0.3%, Nos1+ (by immunohistochemistry), ChAT+ (by GFP), double-negative, double-positive neurons in *ChAT-GCaMP6f* mice. (*C*) Examples of spontaneous activity of cholinergic neurons from *ChAT-GCaMP6f* mice during the period of tonic inhibition, and quantification of these subtypes of spontaneous activity (37 ± 9.4 vs 42.3 ± 14.3 vs 20.7 ± 8.1%, non-active vs sporadically active vs constantly active (c = 122; n = 5 M, 3 F).

To identify the myenteric neuronal substrates underlying spont NgCs, we first evaluated the genetic tools used to drive expression within nitrergic and cholinergic cell types. When we crossed the *Nos1-CreER*^*T2*^ line of transgenic mice, used in this study to express GCaMP6f, to tdTomato-expressing mice as well as to choline acetlyltransferase*-*enhanced green fluorescent protein (*ChAT-eGFP*) transgenic mice, we observed that 90% of neurons labeled with the pan-myenteric neuronal marker Hu expressed either tdTomato or eGFP, consistent with previous reports^23^ (Figure 3*A*). Nearly all Nos1+ cells labeled genetically expressed Nos1 protein by immunohistochemistry, and vice versa (Figure 3*A*). We obtained similar results with the *ChAT-Cre* line together with Nos1 immunostaining (Figure 3*B*). We then imaged cholinergic neurons in the middle colon of *ChAT-GCaMP6f* mice before, during and after spont NgC. During periods of tonic inhibition in between these contractions, many, but not all, cholinergic neurons exhibited Ca^2+^ responses (63% ± 9.4%; c = 122; n = 3 F, 5 M). Some of these neurons exhibited a high number of low-amplitude peaks of fluorescence throughout the period of tonic inhibition and were termed constantly active; others exhibited more sporadic, and typically larger responses, some individual and others with oscillations atop them (Figure 3*C*). The activity of most of these neurons did not appear overtly temporally correlated to themselves or to the small movements associated with phasic contractions. During the spont NgC itself, these responses were replaced by a different kind of Ca^2+^ response described below.

**Figure 4:**
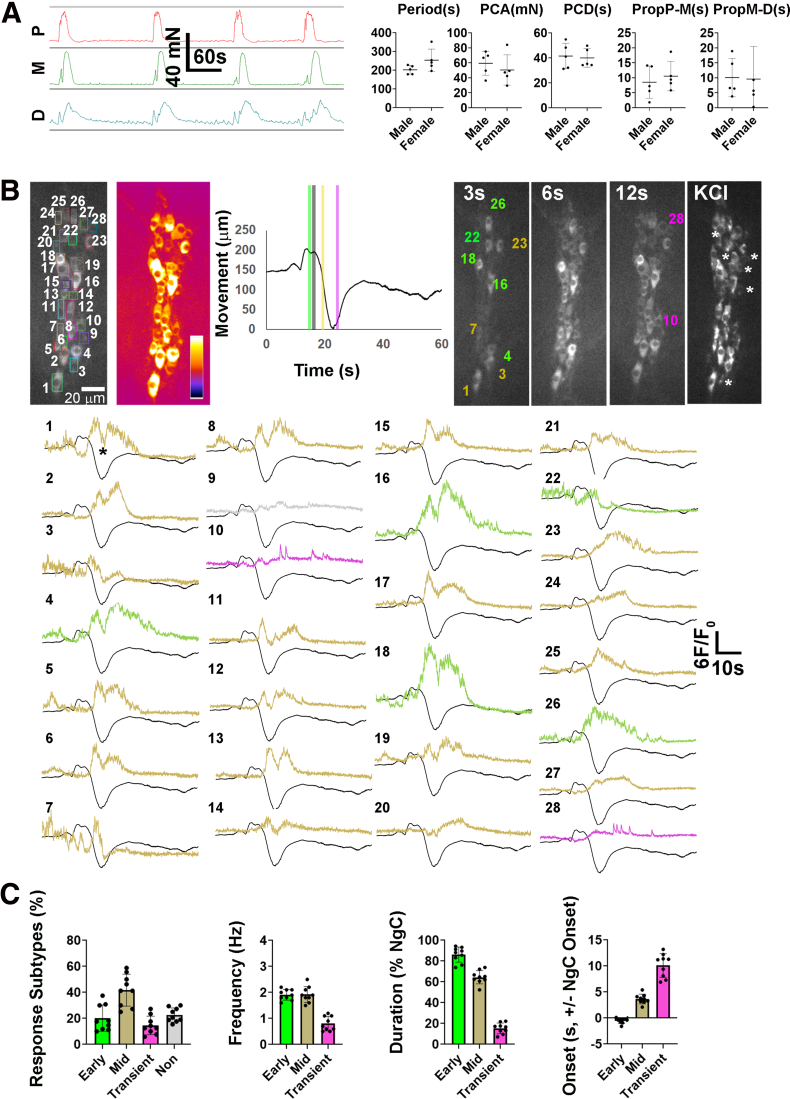
**The Ca**^**2+**^**responses of cholinergic myenteric neurons in the middle colon during Spont NgCs include subtypes with distinct characteristics.** (*A*) *Left*, Representative contractile responses in proximal (*p; red*), middle (*m; green*), and distal (*d; blue*) colon showing spontaneously occurring CMMCs. *Right*, CMMC Period; 1/frequency, amplitude, and duration of the proximal contraction (PCA, PCD), propagation from proximal to middle (Prop P-M) or from middle to distal (Prop M-D) colon were similar between male and female mice (period = 201.9 ± 23.2 vs 253.4 ± 59.3 seconds; *P* = .11; PCA = 59.4 ± 16 vs 50.5 ± 20.2 mN; *P* = .46; PCD = 41.5 ± 10.2 vs 40 ± 7.5 seconds; *P* = .79; Prop P-M = 8.5 ± 5.3 vs 10.5 ± 4.9 seconds; *P* = .55; Prop M-D = 10.1 ± 6.4 vs 9.6 ± 10.8 seconds; *P* = .93; M vs F mice; ∗*P* < .05; Student *t* test; c = 3/n; n = 5 M, 5 F). (*B*) (*Top panel, far left*) Fluorescence image, collected at 20× magnification, of a myenteric ganglion within the middle colon from a *ChAT-GCaMP6f* mouse during a spontaneous neurogenic contraction, with numbered and colored boxes surrounding individual neurons that were selected for analysis of Ca^2+^ transient responses (*lower panel*). (*Top panel, second from left*) Spatial map of the SD of fluorescence intensity (SD map) showing cumulative fluorescence changes in each of the 28 cholinergic neurons identified in the fluorescence image (*Yellow* and *blue* represent 21,000 and 9,000 iu_16_ in fire LUT scalebar). (*Top middle panel*) Movement track during the contraction, measured as displacement in microns (μm) over time. Note the small up and down displacement, likely reflecting a phasic contraction, just before the larger upward displacement, reflecting the initial movement to the right or distal colon, followed by the large downward and subsequent upward displacements, that reflect the local contractile movement (whose onset is represented by *black bar*). *Colored bars* indicate temporal cross-sections of this movement at which the Ca^2+^ response onset of different subpopulations of cells occurs. Three of four images in the *top right panel* show fluorescence images of the ganglion at 2, 6, and 12 seconds after local contractile movement onset, with cell numbers colored according to their response onset: early (*green*), middle (*gold*), and transient (*pink*). The fourth, far-right image in the *top right panel* shows a fluorescence image of the cells in the same ganglion that responded to potassium chloride (KCl), which include a cohort that failed to respond during the spont NgC (*asterisks*). (*Lower panel*), Fluorescence transient responses of cells 1 to 28, plotted together with the movement track in *black*, and color-coded according to their onset as depicted above. For early-responding cells whose responses are green, note that the onset of the response precedes the onset of the downward, local contractile portion of the movement, whereas for those whose responses are gold, note that their onsets occur just after the onset of this movement. Note also that some cells shown in the 2-second image are gold because although their contractile-associated response starts just after the onset of the local contractile movement, they were spontaneously active just before the neurogenic contraction initiated (eg, cells 1,3,7). For cells whose responses are pink, note their transient duration, often late onset, and failure to ride upon a slow rise, in contrast to green-colored and gold-colored responses, which are characterized by small responses riding atop a slow rise. Cell 19 failed to exhibit a response but was tracked, in contrast to the other non-responding cells which were only identified after treatment with KCl; its transient is colored *gray*. Even with motion subtraction, cells may transiently disappear from the plane of focus, which appears as a downward deflection in the middle of the contractile-associated Ca^2+^ response (eg, *asterisk next to response of cell 1*). Y-axis of scalebar represents both displacement in μm and fluorescence in F/F_0_. (*C*) (*Far-left graph*) Relative frequency of the 4 cholinergic response subtypes: early (*green*), middle (*gold*), transient (*pink*) and non (*gray*) responders. (Near-left, near-right, and far-right graphs, respectively) Frequency, duration, and onset, relative to onset of the local contractile movement associated with the neurogenic contraction (NgC), of the responses of the 3 responding cholinergic subtypes. Cellular response subtypes and subtype frequencies, durations, and onsets were not statistically different between males and females (data not shown) and were therefore pooled: 20.2 ± 10 vs 41.7 ± 14.6 vs 14.6 ± 7.1 vs 22.6 ± 5.4 seconds; early vs middle vs transient vs non responders; frequency = 1.9 ± 0.2 vs 1.9 ± 0.3 vs 0.8 ± 0.3 Hz; early vs middle vs transient responders; duration = 86.2 ± 7.3 vs 64.5 ± 6.4 vs 15.4 ± 5.1% NgC length; early vs middle vs transient responders; onset = −0.5 ± 0.5 vs 3.7 ± 0.9 vs 89.0 ± 2.3 seconds; early vs middle vs transient responders; c = 129; n = 4 M, 5 F.

To establish a relationship between spont NgC and the myenteric neuronal Ca^2+^ responses correlated with these contractions (ie, the contractile neuronal substrate), we first evaluated spont NgC by tension recording (CMMC). CMMC frequency, contraction amplitude and duration, and contraction propagation time from proximal to middle to distal colon, were similar between male and female mice (Figure 4*A*). Smaller contractions that initiated in the distal colon and propagated retrogradely were usually observed before the onset of the larger, anterogradely propagating CMMC. Next, we imaged cholinergic neurons within a single ganglion in the middle colon of *ChAT-GCaMP6f* mice before, during and after a spont NgC (Supplementary Video 2; Figure 4*B-C*). Distinct subtypes of Ca^2+^ responses could be observed in cholinergic neurons: one initiated just after the onset of the initial movement, usually to the right, which correlated with the activation of LM described above, and lasted for most of the contractile response. A second began slightly later, at the onset of the larger movement, which correlated with the activation of CM, and also persisted for the majority of the contractile movement. A third subtype was transient and often but not always initiated much later, during the period of smaller movements associated with the peak contractile state (Supplementary Video 2; Figure 4*B–C*). A fourth subtype, identified by response to potassium chloride, failed to exhibit a detectable response during a spont NgC. Each of the first 2 subtypes exhibited oscillations superimposed upon a slower elevation; the frequency of these oscillations was 1.9 ± 0.3 Hz (c = 121; n = 5 M, 4 F). In contrast, the third subtype responses were typically unitary in nature and without oscillations. In some of the first 2 subtypes (eg, cells 1, 3, 5, 7, 8, 19, 22) (Figure 4*B*), but not the second 2 subtypes, small transient responses preceded the larger, longer response that occurred during the contractile movements. Response subtypes were of similar percentage between male and female and therefore were pooled (data not shown). Together, these results demonstrate distinct heterogeneity in the onset, duration, and frequency of activity patterns of cholinergic myenteric neurons during a spontaneous neurogenic contraction. In some of these neurons, smaller responses preceded this contraction.

### Contractile and Cholinergic Responses During Mucosal Stimulation-evoked Contractions

**Figure 5:**
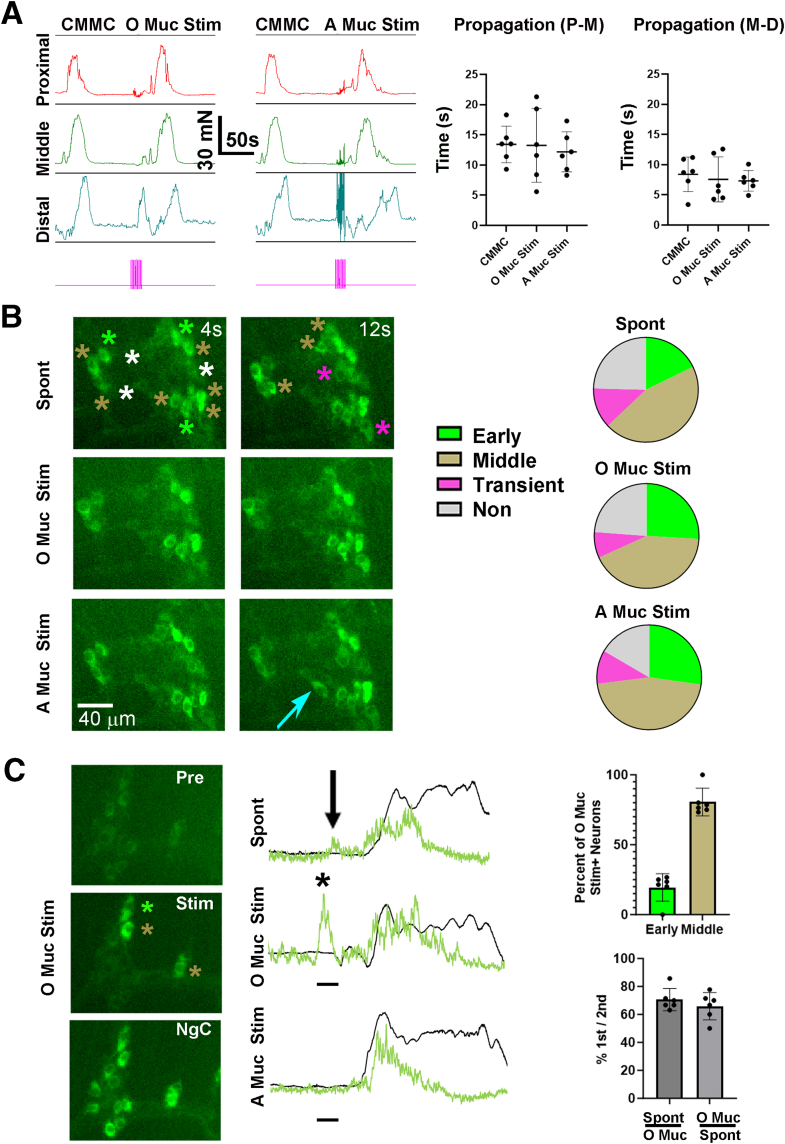
**Shared cholinergic neuronal substrates underlie spontaneous and mucosal stimulation-evoked neurogenic contractions.** (*A*) *Left*, Representative contractile responses in proximal (*red*), middle (*green*), and distal (*blue*) colon during a spont NgC (CMMC) or in response to mucosal stimulation of the proximal end (oral mucosal stimulation or O Muc Stim) or distal end (anal or A Muc Stim) of the colon. *Pink bar* below indicates 10-second period during which stimulation was given. Neither middle colon contraction amplitude nor duration were different (amplitude = 42.6 ± 8.2 vs 44.7 ± 10.6 vs 42.9 ± 9.0 mN; CMMC vs O Muc Stim vs A Muc Stim; *P* = .91; 1-way ANOVA; duration = 42 ± 7.8 vs 43.3 ± 9.7 vs 42.3 ± 7.7 seconds; CMMC vs O Muc Stim vs A Muc Stim; *P* = .96; 1-way ANOVA; c = 6; n = 3 M, 3 F). *Right*, Propagation of the contractions from proximal to middle (Prop P-M) or middle to distal (Prop M-D) colon was also similar between spontaneous and mucosal stimulation-evoked neurogenic contractions (Prop P-M = 13.4 ± 3.0 vs 13.3 ± 6.1 vs 12.2 ± 3.3 seconds; CMMC vs O Muc Stim vs A Muc Stim; *P* = .87; 1-way ANOVA; Prop M-D = 8.4 ± 2.0 vs 7.6 ± 3.7 vs 7.3 ± 1.7 seconds; CMMC vs O Muc Stim vs A Muc Stim; *P* = .23; 1-way ANOVA; c = 6; n = 3 M, 3 F). (*B*) *Left*, Representative images of an individual myenteric ganglion taken at 2 timepoints after the movement onset of spontaneous (*upper panels*), oral/proximal mucosal stimulation-evoked (O Muc Stim; *middle panels*) or anal/distal mucosal stimulation-evoked (A Muc Stim; *lower panels*) neurogenic contractions of the middle colon from a *ChAT-GCaMP6f* mouse. *Green, gold, pink and white asterisks* represent cholinergic response subtypes described in Figure 4. *Right*, Pie charts representing the relative percentage of cholinergic subtypes (early vs middle vs transient vs non = 17.8 ± 14.4 vs 45.2 ± 17.4 vs 12.5 ± 10.1 vs 24.6 ± 7.8%; spontaneous; 25.9 ± 24.6 vs 42.3 ± 22.6 vs 8 ± 8.5 vs 23.8 ± 10%; O Muc Stim; 27.2 ± 25.5 vs 45.8 ± 26.8 vs 10.4 ± 12.2 vs 16.6 ± 13.3%; A Muc Stim; no significant differences in subtype percentage between spontaneous, O Muc Stim, and A Muc Stim by 1-way ANOVA; c = 72; n = 4 M, 3 F). The number of cells exhibiting the same subtype response was 84.5 ± 14.5% between spontaneous and O Muc Stim, 82.6 ± 19.4% between spontaneous and A Muc Stim, and 91.6 ± 9.4% between O and A Muc Stim. (*C*) *Left*, Images of a myenteric ganglion taken before (*pre*), during (*stim*), or during the neurogenic contraction (NgC) caused by O Muc Stim. Note the cells activated by this stimulus, and their identification as early (*green asterisk*) or middle (*gold asterisks*) response subtypes during the ensuing contraction. *Middle*, Transients of the same cholinergic neuron before and during a spontaneous (spont; *top*), oral (O Muc Stim; *middle*) or anal (A Muc Stim; *bottom*) mucosal stimulation-evoked contraction. Note the response during stimulation (*black bar*) of the oral mucosa (*asterisk*) or just before a Spont NgC (*arrow*), but not during stimulation of anal mucosa. *Green trace* indicates this cell is of the early response subtype. *Right*, *Top graph*, of the cholinergic neurons exhibiting a response during O Muc Stim, the percent of them belonging to cholinergic subtypes during the NgC triggered by this stimulation (19.4 ± 9.8 vs 80.6 ± 9.8%; early vs middle; c = 75; n = 3 M, 3 F). *Bottom graph*, of the cholinergic neurons that display a response during O Muc Stim, the percent of them that also exhibit a transient response before a spontaneous NgC stimulation (70.6 ± 8.0%, c = 71; n = 3 M, 3 F); of the cholinergic neurons that display a transient response before a Spont NgC stimulation, the percent of them that also exhibit a response during O Muc Stim (65.8 ± 9.8%; c = 58; n = 3 M, 3 F).

We next examined contractions along the entire colon via tension recording, or cholinergic Ca^2+^ responses in the middle colon via imaging, in response to stimulation of the proximal or distal ends of the colon with a brush. It is well-established that distension induces a contraction immediately proximal to the distending stimulus via the peristaltic reflex,^24^ and distension as a stimulus likely includes distinct sensory components, including stretch of smooth muscle and deformation of mucosa.^25^ Accordingly, in response to mucosal stimulation of the distal colon, a large anterogradely propagating contraction was observed that initiated proximal to the stimulus. However, this contraction initiated at the proximal end of the colon, far from the site of stimulation. Stimulation of the proximal colon also triggered an anterogradely propagating contraction. The amplitude, duration, and propagation times of either of these evoked neurogenic contractions were similar to those occurring during a CMMC (Figure 5*A*). Also similar to CMMC, smaller contractions in the distal colon that often propagated retrogradely were observed in response to mucosal stimulation of either the proximal or distal mucosa, typically just after onset of the stimulation. These distal “pre-contractions” preceded the anterogradely propagating contractions that initiated in the proximal colon (Figure 5*A*).

We next imaged Ca^2+^ responses in the same neurons of the middle colon of *ChAT-GCaMP6f* during spontaneous or evoked contractile movements. We observed large contractions of the middle colon in response to mucosal stimulation of the proximal or distal colon. Interestingly, the onsets of these contractions occurred significantly later after stimulation of the proximal than distal colon (onsets 11.5 ± 5.4 vs 7.9 ± 4.7 seconds after the beginning of stimulation; *P* = .015; c = 51; n = 10 M, 8 F). During these movements, the same neurons that responded during a spont NgC, with the same temporal parameters, were observed during a contraction evoked by each type of mucosal stimulation (Supplementary Videos 3-5; Figure 5*B*). However, in response to stimulation of the proximal but not distal mucosa, a transient, stimulation-locked Ca^2+^ response that preceded the contractile Ca^2+^ response occurred in a subpopulation of cholinergic neurons (31.0% ± 4.7 %; c = 263; n = 4 M, 3 F; 2.16 ± 0.1 Hz; c = 25; n = 3 M, 2 F) (Figure 5*C*). Some of these neurons were also active before spont NgC and belonged to either the early or middle response subtypes during them (100%; *CMMC* = 6; n = 3 M, 3 F) (Figure 5*C*, black arrows and asterisks). This stimulation-locked Ca^2+^ response, as well as the contraction and associated Ca^2+^ responses, were blocked in the presence of hexamethonium (Hex) (100 μM; c = 76; n = 2 M, 3 F), suggesting that they depended on nicotinic synaptic transmission between neurons within the myenteric ganglia. Together, these studies indicate that the same cholinergic neuronal subtypes are active during spontaneous and mucosal stimulation-evoked contractions. In addition, stimulation of the proximal but not distal mucosa triggers a transient Ca^2+^ response in some cholinergic neurons during the stimulation but before the ensuing contraction, and the onset of propagating contractions occurs more slowly after proximal than distal mucosal stimulation.

### Contractile and Cholinergic Responses During Longitudinal Stretch-evoked Contractions

**Figure 6:**
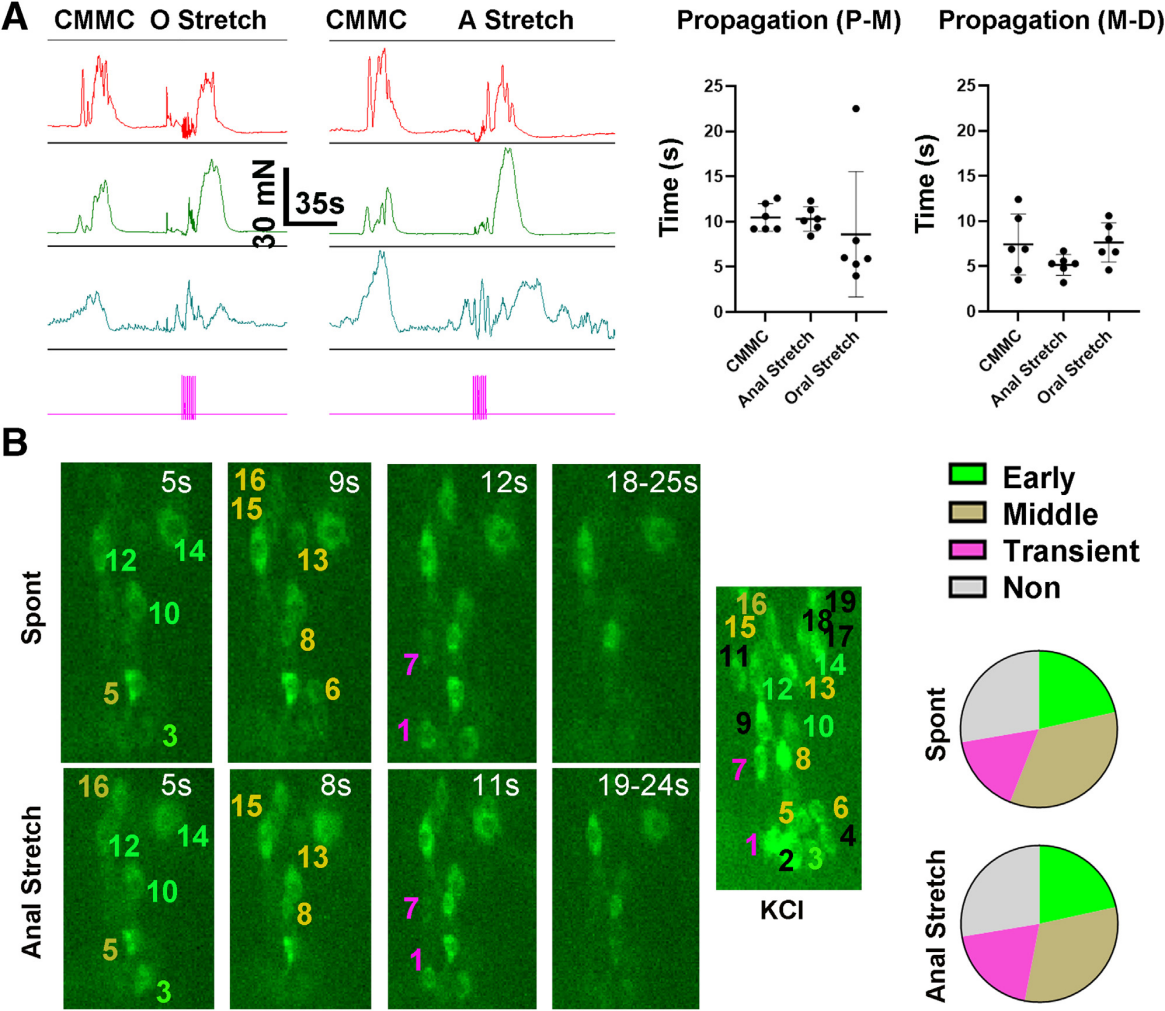
**Shared cholinergic neuronal substrates underlie spontaneous and longitudinal stretch-evoked neurogenic contractions.** (*A*) *Left*, Representative contractile responses in proximal (*red*), middle (*green*), and distal (*blue*) colon during a CMMC or in response to longitudinal stretch of the proximal or distal end of the colon (oral, anal stretch). Both oral and anal stretch evoked a contraction of the middle colon with similar amplitude and duration to that of CMMC (Amplitude = 49.6 ± 21.3 vs 53 ± 13.7 vs 43.5 ± 10.6 mN; CMMC vs anal stretch vs oral stretch; *P* = .58; 1-way ANOVA; Duration = 41 ± 6.2 vs 34 ± 9.3 vs 35.9 ± 9.6 seconds; CMMC vs anal stretch vs oral stretch; *P* = .37; 1-way ANOVA; c *=* 6; n = 2 M, 3 F). *Right*, Propagation of the contractions also similar between spontaneous and oral and anal stretch-evoked neurogenic contractions (Prop P-M = 10.5 ± 1.5 vs 10.3 ± 1.4 vs 8.6 ± 6.9 seconds; CMMC vs anal stretch vs oral stretch; *P* = .7; 1-way ANOVA; Prop M-D = 7.4 ± 3.4 vs 5.2 ± 1.1 vs 7.6 ± 2.2 seconds; CMMC vs anal stretch vs oral stretch; *P* = .17; 1-way ANOVA; c = 6; n = 3 M, 3 F). (*B*) *Left*, Representative images of an individual myenteric ganglion taken at several timepoints after the movement onset of spontaneous (*upper panels*; spont) or anal mucosal stimulation-evoked (*lower panels*; anal stretch) neurogenic contractions of the middle colon from a *ChAT-GCaMP6f* mouse. *Green, gold, pink and white asterisks* represent cholinergic response subtypes described in Figure 4, Figure 5. *Right*, Pie charts representing the relative percentage of cholinergic subtypes (early vs middle vs transient vs non = 21.5 ± 4.5 vs 34.5 ± 5.4 vs 16.3 ± 3.4 vs 27.6 ± 4.6%; spontaneous; 21.6 ± 5 vs 31.4 ± 5.4 vs 19.4 ± 3.5 vs 27.6 ± 4.6%, anal stretch; No significant differences in subtype percentage between spontaneous and A Stretch by Student *t*-test; c = 66; n = 2 M, 2 F). The number of cells exhibiting the same subtype response was 89.5 ± 2.6% between spontaneous and anal stretch (for example, cell 6 responds as a middle subtype during a spontaneous neurogenic contraction but is a non-responder during anal stretch).

We then tested whether other methods of stimulation commonly used to evoke neurogenic contractions resulted in similar patterns of propagating contractions throughout the colon by tension recording and cholinergic Ca^2+^ responses in the middle colon by imaging. Elongation of the distal colon increases fecal pellet propulsion and thus peristaltic activity^26^ and induces an immediate propagating neurogenic contraction,^27^ whereas elongation of proximal colon reduces pellet transit and fails to induce a contraction.^26^^,^^27^ However, in response to a 5% to 10% longitudinal stretch of the colon initiated at either side, anterogradely propagating contractions were observed (n = 2 M, 3 F). The amplitude, duration and propagation of these contractions, were similar to those during a CMMC (Figure 6*A*). Similar to mucosal stimulation, longitudinal stretch initiated at either end of the colon initiated a smaller distal pre-contraction during the stimulus that propagated retrogradely before the onset of the larger, anterogradely propagating contractions that originated in proximal colon.

Next, we imaged Ca^2+^ responses in cholinergic neurons of the middle colon during a spontaneous or longitudinal stretch-evoked neurogenic contraction. Similar to mucosal stimulation, longitudinal stretch of the proximal colon initiated a contraction whose onset was later than that induced by stretch of distal colon (onsets 10.9 ± 4 vs. 5.8 ± 2.2 seconds after the beginning of proximal vs. distal stimulation; *P* = .0004; c = 26; n = 8 M, 4 F). Also similar to mucosal stimulation, longitudinal stretch of either end of the colon produced a response during the evoked contraction in the same distinct cholinergic subtypes that were activated during a spont NgC (Figure 6*B*; data not shown). Finally, and also similar to mucosal stimulation, stretch of the proximal, but not distal colon, activated 28.1% ± 12% (n = 2 M, 2 F) of cholinergic neurons during the stimulus, a response that was blocked by Hex (n = 2 M, 2 F). These results demonstrate that longitudinal stretch initiated at either end of the colon, similar to mucosal stimulation, triggers a distal contraction followed by a proximo-distal propagating contraction that utilizes the same neuronal substrate as spont NgC. In addition, longitudinal stretch, similar to mucosal stimulation, of proximal but not distal colon, triggered a distinct Ca^2+^ response in a subtype of cholinergic neurons in the middle colon during the stimulation, and produced subsequent contractions with a later onset than those evoked by distal stretch or mucosal stimulation.

### Contractile and Cholinergic Response Patterns During Direct and Indirect Electrical Stimulation-evoked Contractions

**Figure 7:**
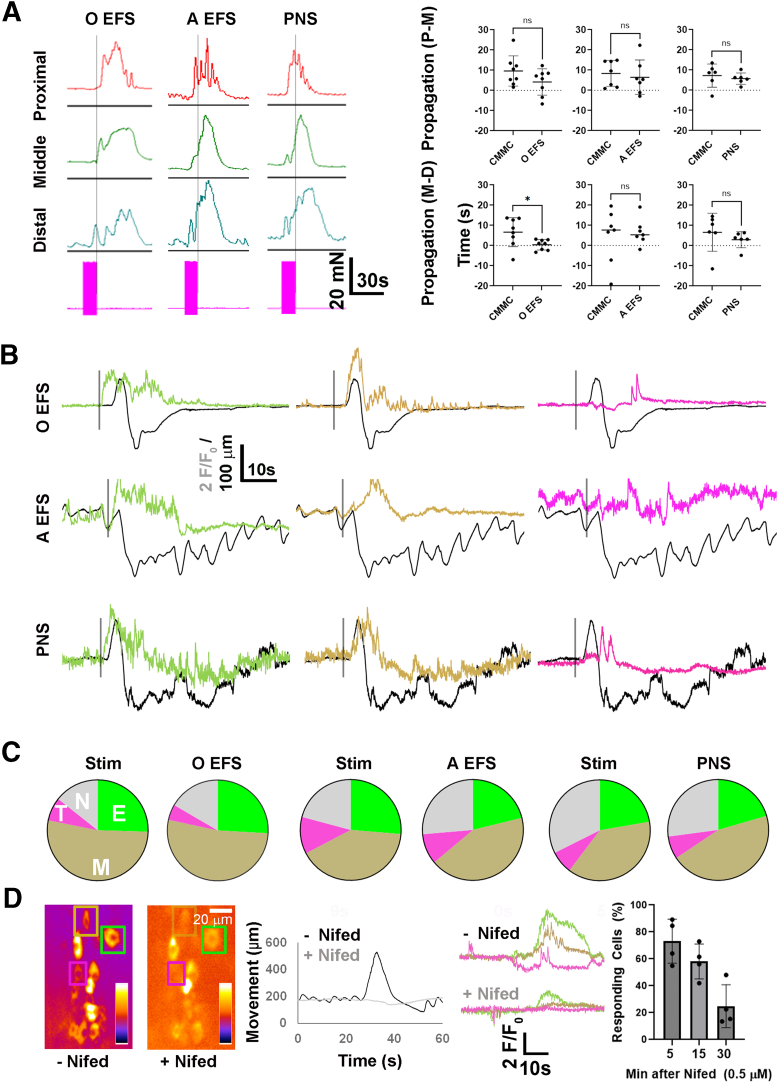
**Distinction between the contractile pattern within, and cholinergic neuronal substrates underlying, spontaneous and electrically evoked neurogenic contractions.** (*A*) *Left*, Representative contractile responses in proximal (*red*), middle (*green*), and distal (*blue*) colon in response to 10 seconds of 20 Hz direct EFS of the proximal colon (oral or O EFS), or distal colon (anal or A EFS), or indirect stimulation of the pelvic nerve (PNS). *Pink box* marks the period of stimulation, and *black vertical bars* indicate the cessation of stimulation. Direct EFS of proximal or distal colon, or PNS, evoked a contraction of the middle colon with similar amplitude and duration to that of CMMC (amplitude = 46.2 ± 10 vs 52.3 ± 22.6 vs 47.3 ± 13.1 vs 48.4 ± 14.5 mN; CMMC vs O EFS vs A EFS vs PNS; *P* = .97; 1-way ANOVA; duration = 39.7 ± 6.4 vs 31.7 ± 15.1 vs 35.1 ± 10.2 vs 40.1 ± 10 seconds; CMMC vs O EFS vs A EFS vs PNS; *P* = .40; 1-way ANOVA; c = 6; n = 2 M, 3 F). *Right*, Direct oral EFS, but not anal EFS or PNS, elicited neurogenic contractions with significantly faster propagation speeds from middle to distal colon (peak-to-peak proximal-middle [P-M] duration = 9.7 ± 7.5 vs 4.2 ± 6.5 seconds; CMMC vs O EFS; *P* = .14; c = 8; n = 5 M, 3 F; 8.3 ± 6.5 vs 6.5 ± 8.5 seconds; CMMC vs A EFS; *P* = .65; c = 6; n = 3 M, 3 F; 7.3 ± 5.7 vs 5.8 ± 2.6 seconds; CMMC vs PNS; *P* = .57; c = 6; n = 3 M, 3 F; peak-to-peak middle-distal [M-D] duration = 6.7 ± 7.2 vs 0.4 ± 2.5; CMMC vs O EFS; *P* = .036; 4.7 ± 13.4 vs 6.7 ± 6.5; CMMC vs A EFS; *P* = .73; 6.6 ± 9.4 vs 3.0 ± 6.3; CMMC vs PNS; *P* = .40; Student *t* test). Beginning-to-beginning and end-to-end M-D, but not P-D, contractile durations were also significantly longer in response to O EFS but not to A EFS or PNS. The time to pre-contraction onset (2.5 ± 0.9 vs 8.2 ± 2.8 seconds; *P* = .0004) was faster in response to PNS than to O EFS, but not to A EFS (2.5 ± 0.9 vs 3.91 ± 2.1 seconds; *P* = .1654; c = 14; n = 8 M, 6 F; ∗*P* < .05, Student *t* test with Bonferroni correction). (*B*) Representative cholinergic Ca^2+^ response transients, together with movement trajectories, of neurogenic contractions triggered by oral or anal EFS or PNS, whose onset is indicated in each trace pair by *vertical black bar*. In response to each stimulation, early responding neurons (green traces) exhibit a Ca^2+^ response that precedes movement onset, whereas middle and transient neuronal subtypes (*gold and pink traces*) initiate a response after movement onset. Note that Ca^2+^ responses to O EFS were only categorized if the contraction initiated during the stimulation (4 of 16 cases). (*C*) Pie charts representing the relative percentage of cholinergic subtypes (Early (E) vs middle (M) vs transient (T) vs non (N) = 25.5 ± 18.4 vs 52.7 ± 9.6 vs 7.5 ± 15 vs 14.3 ± 5.6%; A Muc Stim; 25.9 ± 11.3 vs 52.5 ± 13.1 vs 5 ± 5.8 vs 16.6 ± 7.4; O EFS; No significant differences in subtype percentage between spontaneous and O EFS by Student *t*-test; c = 42; n = 1 M, 3 F. Early vs middle vs transient vs non = 23.6 ± 4.2 vs 42.5 ± 10.3 vs 12.3 ± 9.7 vs 21.6 ± 5.7%; A Muc Stim; 21.1 ± 8.2 vs 42.7 ± 8.1 vs 9.8 ± 8.2 vs 26.4 ± 5.6%, A EFS; No significant differences in subtype percentage between spontaneous and A EFS by Student *t*-test; c = 42; n = 2 M, 2 F; Early vs middle vs transient vs non = 22.3 ± 5.6 vs 38 ± 10.4 vs 7.3 ± 8.5 vs 32.4 ± 11.7%; A Muc Stim; 20.5 ± 6.6 vs 45.1 ± 4.5 vs 7.3 ± 8.5 vs 27.1 ± 8.9%, PNS; No significant differences in subtype percentage between spontaneous and PNS by Student *t*-test; c = 49; n = 3 M, 1 F). The number of cells exhibiting the same subtype response was 84.2 ± 5.8% between A Muc Stim and O EFS, 93.3 ± 5.8% between A Muc Stim and A EFS, and 89.3 ± 4.8% between anal mucosal stimulation and PNS. (*D*) *Far left*, SD Maps of cholinergic neuronal responses to A Muc Stim before (− Nifed) and 5 minutes after (+ Nifed) perfusion of nifedipine (0.5 μM in EtOH). *Yellow and blue* represent 16,000 and 12,000 iu_16_ in fire LUT scalebar in the absence of nifedipine, and 12,000 and 10,000 iu_16_ in the presence of nifedipine. *Near left*, movement traces during neurogenic contraction induced by A Muc Stim before (*black trace*, − Nifed) or 5 minutes after (*gray trace*, + Nifed) perfusion of nifedipine; note absence of phasic contractions, and near complete absence of neurogenic contraction, after nifedipine. *Near right*, Ca^2+^ responses of 3 cholinergic neurons exhibiting early (*green*), middle (*gold*), and transient (*pink*) subtype onsets, before (− Nifed) or 5 minutes after (+ Nifed) nifedipine. *Far right*, Number of responding cholinergic neurons in response to A Muc Stim in the presence of nifedipine for 5, 15, and 30 minutes, as a percentage of the same neurons responding to A Muc Stim in the absence of nifedipine (73.2 ± 16.4 % after 5 minutes, vs 58.1 ± 12.9 after 15 minutes, vs 24.9 ± 15.9 % after 30 minutes; c = 106; n = 2 M, 2 F.

We then examined the response of cholinergic neurons to direct EFS, a stimulus used to study the nature of postsynaptic junctional potentials caused by the release of excitatory or inhibitory transmitter in strips of gut tissue,^28^ and which produces propagating contractions in whole colon.^29^^,^^30^ Similar to mucosal stimulation or longitudinal stretch, transmural EFS delivered to either proximal or distal colon (10 seconds of 20 Hz) produced anterogradely propagating contractions with similar amplitudes and durations of those during CMMC (n = 4 M, 4 F). However, EFS of the proximal colon produced a significantly shorter propagation from the middle to distal colon (Figure 7*A*). EFS also produced a distal pre-contraction whose onset was later when delivered to proximal than to distal colon (8.2 ± 2.8 s vs 3.9 ± 2.1; *P* = .006; c = 15; n = 3 M, 3 F). Similarly, the onset of the ensuing anterogradely propagating contractions occurred later in response to EFS of proximal than that of distal colon (16.1 ± 6 s vs 10.6 ± 2.6 s; *P* = .043; c = 15; n = 3 M, 3 F) (Figure 7*A*).

In response to EFS of proximal colon, large contractile-associated movements were observed in the middle colon of imaging preparations in 15 of 16 cases. The onset of these movements varied from a few seconds after EFS onset to just after EFS cessation. In every case, Ca^2+^ responses were observed in some but not all cholinergic neurons rapidly after EFS onset (0.88 ± 0.12 s; c = 31; n = 3 M, 3 F). The majority of cells that responded quickly after the onset of EFS of the proximal colon, before the ensuing contraction, also responded during mucosal stimulation or stretch of the proximal colon before the ensuing contraction (74.5 ± 8.4%; c = 83; n = 2 M, 3 F). In response to EFS of the proximal colon, the relationship between Ca^2+^ responses and EFS-induced neurogenic contractile movements depended on whether these movements occurred during or just after the cessation of EFS. If the movement occurred before, Ca^2+^ responses occurred in the same temporal subtypes as in response to a spont NgC (4 of 16) (Figure 7*B, C*). If the movement occurred just after, many of the cholinergic Ca^2+^ responses that normally occurred only after movement onset actually preceded it and then persisted for variable lengths of time during the movement (11 of 16). By contrast, in response to EFS of the distal colon, large contractile responses occurred more rapidly in the middle colon (onsets 6.7 ± 2.3 vs 10.4 ± 2.7 seconds after the beginning of distal vs proximal EFS; *P* = .006; c = 26; n = 8 M, 13 F), similar to the effects of mucosal stimulation and stretch of proximal vs distal colon. Ca^2+^ responses were observed in a small subset of cholinergic neurons rapidly after EFS onset, and these neurons exhibited the early response subtype during the ensuing contraction, whereas middle-, transient-, and non-responders were observed only during the contraction (Figure 7*B, C*).

Finally, we investigated the effect of indirect EFS via stimulation of the pelvic nerve (PNS), which evokes a pan-colonic peristaltic reflex and enhances fecal pellet propulsion.^26^^,^^31^^,^^32^ Ten seconds of 20 Hz of PNS triggered a propagating neurogenic contraction when measured by tension recording, the contractions of which were similar in magnitude, duration, and propagation time as spont NgC (Figure 7*A*). PNS also induced a distal pre-contraction (Figure 7*A*). Similar to the effects of distal direct EFS, PNS triggered a Ca^2+^ response within 1 second in the early response subtype of cholinergic neurons, whose onset preceded contractile movement onset, followed by response subtypes that occurred during the movement in similar frequencies as in spont NgC (Figure 7*B, C*). Thus, PNS elicits the same neuronal contractile substrate as that produced by other forms of distal colonic stimulation.

Large, neurogenic contractile movements often made active cells disappear from the x, y, or z planes and thus precluded their analysis. We noted that many studies measuring the electrical activity underlying the CMMC (eg, Bywater et al^8^) as well as those measuring Ca^2+^ responses during the CMMC in chemical dye-loaded preparations^18^ utilized the paralytic properties of dihydropyridines, which reduce movement of the colon by blocking Ca^2+^ influx through L-type voltage-gated calcium channels (VGCC) expressed by CM/LM cells. However, even at low concentrations, which reduce but do not completely block movement (eg, 0.5 μM nifedipine), the presence of these drugs quickly (within 10 minutes) reduced both the number of responding cholinergic neurons as well as the response amplitudes in those that did respond during evoked neurogenic contractions (Figure 7*D*). These results are consistent with previous studies,^33^^,^^34^ which found that spontaneous activity of VGCC-expressing myenteric neurons in the adult mouse colon was abolished after treatment with nicardipine.

### Nitrergic Response Patterns During Spontaneous and Evoked Colonic Contractions

**Figure 8:**
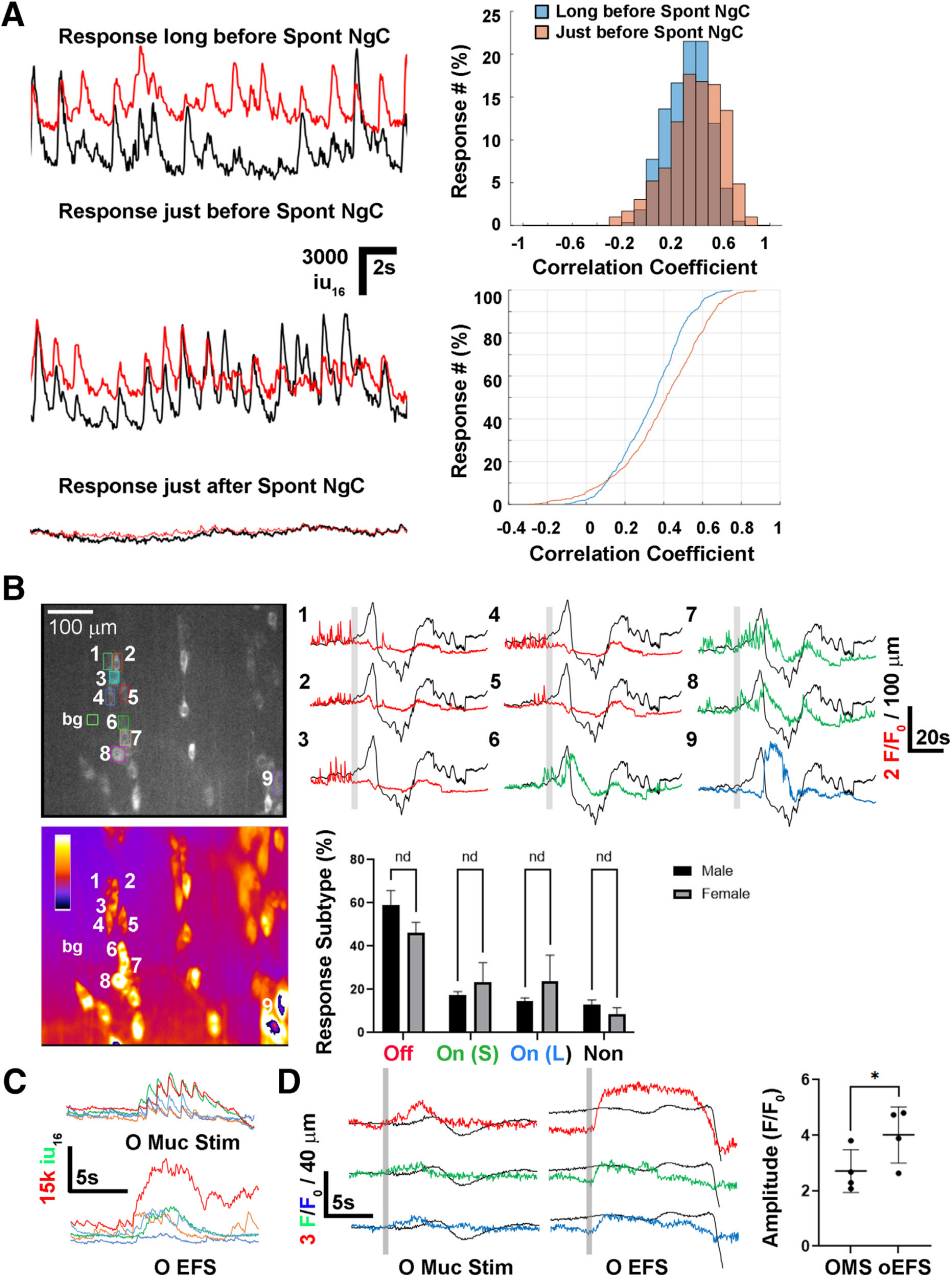
**The Ca**^**2+**^**responses of nitrergic myenteric neurons in the middle colon before, during and after spontaneous and evoked neurogenic contractions include subtypes with distinct characteristics.** (*A*) *Left*, Nearly all nitrergic neurons (90.8 ± 3.8%; c = 418; n = 3 M, 4 F) exhibited “tonic,” low-amplitude Ca^2+^ responses with a similar frequency (0.83 ± 0.08 Hz; c = 120; n = 2 M, 2 F). Representative Ca^2+^ responses of the same 2 cells at least 1 minute before the onset of a spont NgC (*top*), immediately before a spont NgC (*middle*), and immediately after a spont NgC (*bottom*). The average frequency (0.82 ± 0.06 vs 0.84 ± 0.09 Hz, long before vs just before spont NgC; *P* = .68; Student’s *t* test) and amplitude (1756 ± 372 vs 2288 ± 1630 iu_16_, long before vs just before spont NgC; *P* = .47; Student’s *t* test) were unchanged (c = 120; n = 2 M, 2 F). In contrast, the temporal coordination (KS2 stat = 0.18, long before vs just before spont NgC; *P* = .0012, Kolmogorov-Smirnov test; c = 168, all tested pairwise to each other; n = 2 M, 2 F) was enhanced just before a spont NgC. *Right*, From a representative experiment, a frequency distribution of the correlation coefficients of 37 cells compared with each of themselves, long before or just immediately before a spont NgC, is plotted. *Light blue* represents values from long before a spont NgC, *light brown* values from immediately before a spont NgC, and *dark brown* the overlap. A cumulative frequency distribution of these correlation coefficients is plotted below; *blue line* indicates distribution long before, and *red line* indicated distribution immediately before, a spont NgC. Note the shift to the right from blue to red, a change represented by a vertical line drawn between these two distributions at their greatest gap and designated as the KS2 statistic. (*B*), *Left*, Representative fluorescence image (*upper*) or SD map (*lower*) of myenteric ganglia from the middle colon of a *Nos1-GCaMP6f* mouse. *Right upper*, movement track (*black*) or Ca^2+^ response transients (*colored*) from numbered nitrergic neurons within the ganglia, showing the cessation of low-amplitude responses (“off” response; peak amplitude average = 1.08 ± 0.26 F/F_0_) that characterize the period of tonic inhibition in between spont NgC (Cells 1–5; *red*), or the initiation of short- (Cells 6–8; *green*) or long-duration (Cell 9; *blue*) large-amplitude responses just after the onset of a spont NgC (“on” response; peak amplitude average = 3.06 ± 0.66 F/F_0_; n = 3 M, 3 F); *gray bars* = movement onset. The enhanced intensity of the “on” vs. “off” responses are also depicted by the SD map, whose signal tracks the cumulative fluorescence intensity of the response, rather than the peak amplitude (ie, compare brightness of cells 1–5 vs 6–9). The percentage of cells exhibiting each of these patterns of activity (58.9 ± 6.8 vs 46.2 ± 4.8%; “off,” male vs female; 17.3 ± 1.6 vs 23.5 ± 8.9%; “short on,” male vs female; 14.5 ± 1.7 vs 24 ± 12%; “long on,” male vs female; 13 ± 2.2 vs 8.7 ± 2.9% “non-responders,” male vs. female) are statistically similar (no different; nd) between nitrergic neurons of male and female mice (c = 177; n = 3 M, 3 F). (*C*) Representative Ca^2+^ responses from nitrergic neurons during mucosal stimulation of the proximal colon (O Muc Stim; *upper image*) or during EFS of the proximal colon (O EFS; *lower image*). Note the synchronization of nitrergic neurons that occurs during O Muc Stim, similar to that which occurs just prior to a spont NgC. Because the stimulation frequency is at 20 Hz, this synchronization is difficult to observe in response to O EFS. (*D*) Left, responses of the same 3 nitrergic neurons to either O Muc Stim or O EFS. *Gray bars* show stimulation onset. *Right*, the amplitude of nitrergic Ca^2+^ responses to O Muc Stim is less than that of those to O EFS (2.71 ± 0.77 vs 4.01 ± 1.0 F/F_0_; c = 59; n = 1 F, 3 M). Nearly all nitrergic cells examined in the middle colon exhibited a response to EFS (98 ± 3.7 vs 85.7 ± 13.4% responsive to proximal mucosal stim vs proximal EFS; *P* = .018; c = 211; n = 10 M, 5 F). Response area under the curve (AUC) was not performed since the duration of O Muc Stim is about one-half that of O EFS.

We next imaged nitrergic neurons in the middle colon of *Nos1-GCaMP6f* mice before, during and after spont NgCs. During the period of tonic inhibition before such a contraction, nearly all nitrergic neurons exhibited “tonic,” low-amplitude Ca^2+^ responses with a similar frequency, which increased in temporal coordination the closer it was to the onset of a spont NgC (Supplementary Videos 6-7; Figure 8*A*). During this contraction, which was characterized by a different kind of Ca^2+^ response described below, these low-amplitude Ca^2+^ responses were not observed and did not return until after the cessation of the contraction (Figure 8*A*). Together, these data show that nitrergic neurons exhibit a roughly 1 Hz activity pattern during the tonic inhibitory period, and that the synchrony of this activity is enhanced immediately prior to a spont NgC, at which point a large population of these neurons then ceases their activity.

Nitrergic neurons of the middle colon also exhibited distinct Ca^2+^ response subtypes during a spont NgC (Figure 8*B*). The first one, affecting 52.6% ± 8.8% of these neurons (c = 177; n = 3 F, 3 M), exhibited a marked cessation of the low-amplitude activity pattern described above, which lasted throughout the contraction, and are hence referred to as “off” nitrergic neurons. The second response subtype, affecting 39.6% ± 7.7% of nitrergic neurons, was composed of a large increase of fluorescence, atop which oscillations were observed, that initiated just after the onset of the larger movement, which correlated with the activation of CM in *CAGGS-GCaMP3* mice. Within this “on” nitrergic response subtype, an additional subdivision was noted: one that was more transient, lasting 10 to 12 seconds (eg, cells 6–8 in Figure 8*B*), and one that persisted the remainder of the contraction (eg, cell 9 in Figure 8*B*). This latter, “long on” subtype was noticeably larger in size than each of the other 2 nitrergic subtypes (somal area = 566.8 ± 65.8 μm^2^; n = 2 M, 3 F).

To determine if this nitrergic contractile substrate was also observed during evoked contractions, we stimulated proximal mucosa with a brush. The percentages of “off” and “on” nitrergic response subtypes were similar to those during a spont NgC. Interestingly, most nitrergic neurons also exhibited a hex-sensitive, synchronized Ca^2+^ response of about 1 Hz during mucosal stimulation of proximal but not distal colon (85.7 ± 13.4%; c = 138; n = 3 F, 5 M) (Supplementary Videos 8–9; Figure 8*C*). The response of nitrergic neurons to direct EFS of the proximal colon was similar as the response to proximal mucosal stimulation, except that the response was larger to EFS (Figure 8*D*). Together, these studies show that, similar to cholinergic neurons, nitrergic neurons of the middle colon exhibit a common contractile substrate associated with spontaneous and evoked contractions. Additionally, most nitrergic neurons exhibit a synchronized response to stimulation of proximal, but not distal colon, a response also observed just prior to a spont NgC.

### Nitrergic Neurons but not NO is Required for Propagation of CMMC

**Figure 9:**
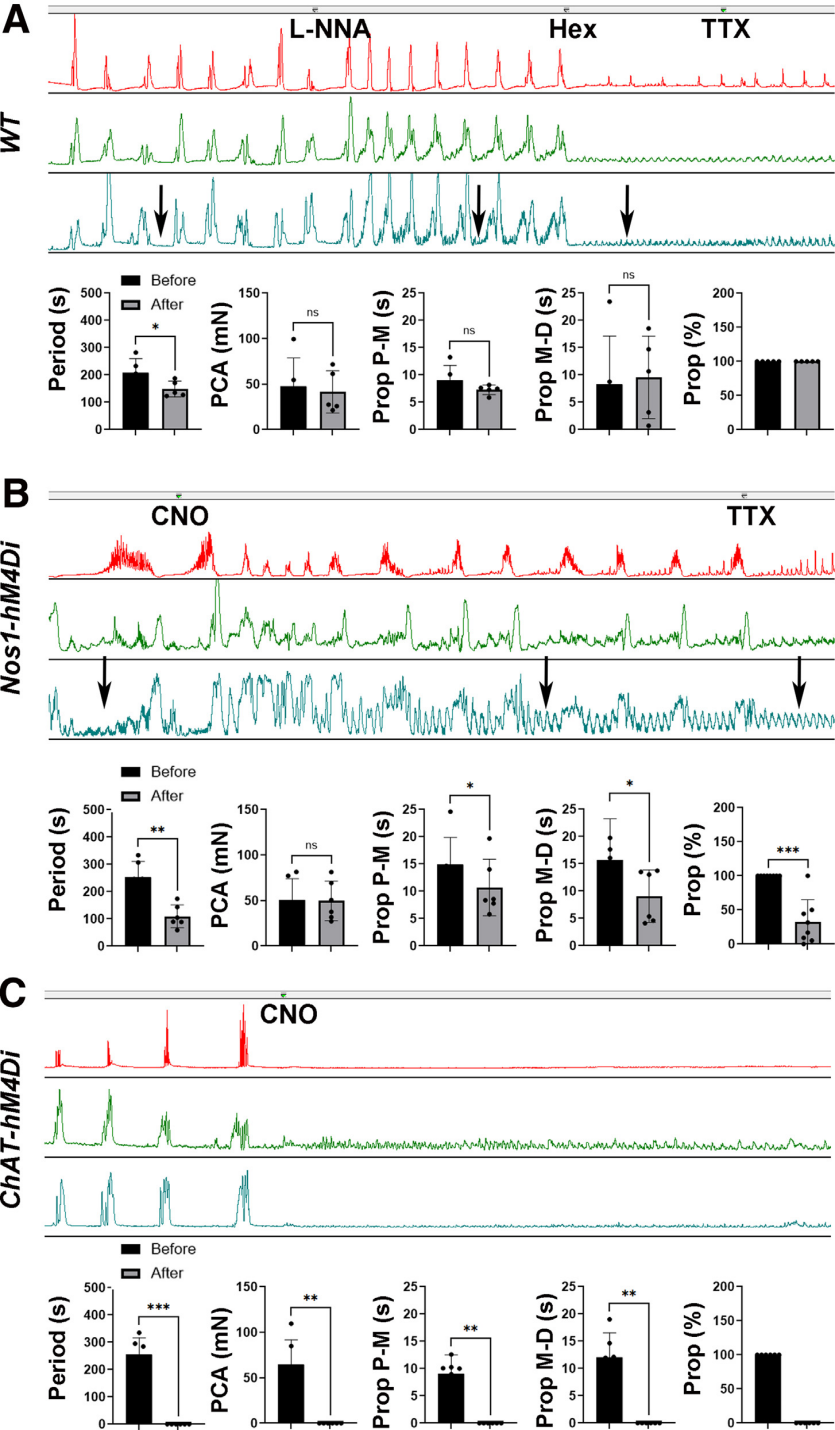
**Nitrergic neuronal activity but not nitric oxide is required for CMMC propagation.** (*A*) Representative contractile responses in proximal (*red*), middle (*green*), and distal (*blue*) colon during CMMC before or after the addition of the NO synthesis inhibitor L-NNA (100 μM; perfused at first small triangle in gray bar above contractile recordings). The frequency of CMMC was significantly increased with no effect on proximal contractile amplitude (PCA) or propagation times or percentage (period = every 207.6 ± 52.5 vs every 148.84 ± 28.91 seconds; *P* = .013; PCA = 47.3 ± 31.5 vs 41.5 ± 23.2 mN; *P* = .37; Prop P-M = 9 ± 2.7 vs 7.3 ± 0.9 seconds; *P* = .25; Prop M-D = 8.3 ± 8.9 vs 9.6 ± 7.6 seconds; *P* = .7; Prop % = 100 vs 100%; pre- vs post-L-NNA; c = 25; n = 2 M, 3 F; ∗*P* < .05; Student *t* test). Hex (100 mM) and then TTX were then added to eliminate neurogenic contractions. *Arrows* point to myogenic contractions in distal colon before and after drugs. (*B*) The frequency of CMMC was significantly increased, and propagation times and percentage were significantly affected, with no effect on proximal contractile amplitude, after chemogenetic silencing of nitrergic neurons with CNO (period = every 252.8 ± 58.4 vs every 109.9 ± 41.7 seconds; *P* = .008; PCA = 50.5 ± 23.4 vs 49.6 ± 21.7 mN; *P* = .8; Prop P-M = 14.9 ± 5 vs 10.7 ± 5.2 seconds; *P* = .025; Prop M-D = 15.7 ± 7.5 vs 9.1 ± 4.8 seconds; *P* = .047; Prop % = 100 vs 34.3%; pre- vs post-CNO; c = 30; n = 3 M, 3 F; ∗*P* < .05; Student *t* test). Propagation of contractions from middle to distal colon was more severely affected than from proximal to middle colon. CMMC frequency was reduced 1.4-fold by L-NNA and 2.7-fold by nitrergic silencing. Note that the increase of CMMC frequency after L-NNA as well as nitrergic silencing is most pronounced in the first 10 minutes after drug treatment. *Arrows* point to myogenic contractions in distal colon before and after drugs. (*C*) CMMC are abolished after chemogenetic silencing of cholinergic neurons with CNO (period = every 255.4 ± 61.1 vs 0 seconds; *P* = .0002; PCA = 64.5 ± 27.3 vs 0 mN; *P* = .002; Prop P-M = 9 ± 3.5 vs 0 seconds; *P* = .0015; Prop M-D = 12 ± 4.5 vs 0 seconds; *P* = .0013; Prop % = 100 vs 0 %; pre- vs post-CNO; c = 30; n = 3 M, 3 F; ∗*P* < .05; Student *t* test).

The temporal coordination of nitrergic neurons just before a spont NgC, coupled with their abrupt cessation of activity, suggests a role for these cells in mediating the pre-complex hyperpolarization that precedes these contractions, and possibly the ensuing depolarization that follows. In other words, the activity of these cells, some of which are inhibitory motor neurons projecting to muscle, may be required for the generation of CMMC. Although many studies report that treatment with the NO synthesis inhibitor L-N^G^-nitroarginine (L-NNA) not only fails to stop the generation of CMMC, but actually enhances their frequency,^12^^,^^14^^,^^15^^,^^35^ a recent study argues that NO is required for these contractions.^17^ However, we found that L-NNA enhanced the frequency of CMMC without reducing their amplitude, duration or propagation times (Figure 9*A*), suggesting that NO is dispensable for CMMC generation. It is worth noting that these effects on CMMC frequency of L-NNA were strongest just after perfusion (CMMC every 205.4 ± 53.8 vs 121.2 ± 20.3 seconds, before vs after first 10 minutes of L-NNA perfusion; n = 2M, 3F). In addition to increasing the frequency of neurogenic contractions, L-NNA also increased the amplitude and frequency of TTX-resistant myogenic contractions. To test whether substances within nitrergic neurons other than NO are required for CMMC, we treated *Nos1-hM4Di* mice with clozapine n-oxide (CNO; 10 μM) to silence them. In the proximal colon, contractions were unaffected in amplitude and duration and enhanced in their frequency, similar to treatment with L-NNA. In some samples (2 of 8), propagation of these contractions was unaffected (Figure 9*B*). In other samples, the number of middle and distal contractions that propagated was reduced (6 of 8). Similar to L-NNA, the amplitude and frequency of myogenic contractions was markedly increased. Thus, substances within nitrergic neurons are not required for contractions observed in proximal colon, but likely facilitate propagation of these contractions throughout the colon. By contrast, CNO-treated *ChAT-hM4Di* mice exhibited a complete shutdown of CMMC in all regions of colon (Figure 9*C*). However, these effects are likely mediated by central changes within the ganglia, mediated by nicotinic receptors, as well as by peripheral changes at the neuroeffector junction, mediated by muscarinic receptors.

### Molecular Characterization of Cholinergic and Nitrergic Subtypes Identified by Activity Patterns During Neurogenic Contractions

The current study identified distinct subpopulations of cholinergic and nitrergic myenteric neurons based on patterns of activity during neurogenic contractions. However, the identity of these neuronal subtypes remains unclear. Various methods can be used to identify subtypes of myenteric neurons, including morphologic, molecular, functional, and tract tracing techniques. We chose to determine whether subtypes described in our study corresponded to the molecular subclasses identified in recent transcriptomic studies of myenteric neurons.^36^, ^37^, ^38^, ^39^, ^40^ We focused on candidates that are reportedly restricted to individual molecular subclasses, such as somatostatin (Sst) and glutamic acid decarboxylase 2 (GAD2).^38^^,^^39^ The Sst+ subclass includes features of intrinsic primary afferent neurons (IPANs) as well as descending interneurons (INs), based on transcriptomic studies (IPAN class PSN4 in Drokhlyansky et al^36^ IPAN class B3 in Elmentaite et al^37^ IN class 5 in Morarch et al^39^ IPAN/IN cluster 7 in May-Zhang et al^38^), as well as morphologic and functional studies,^41^, ^42^, ^43^, ^44^ and are cholinergic. The GAD2+ subtype may represent a nitrergic and cholinergic subclass of descending IN.^38^^,^^39^

**Figure 10:**
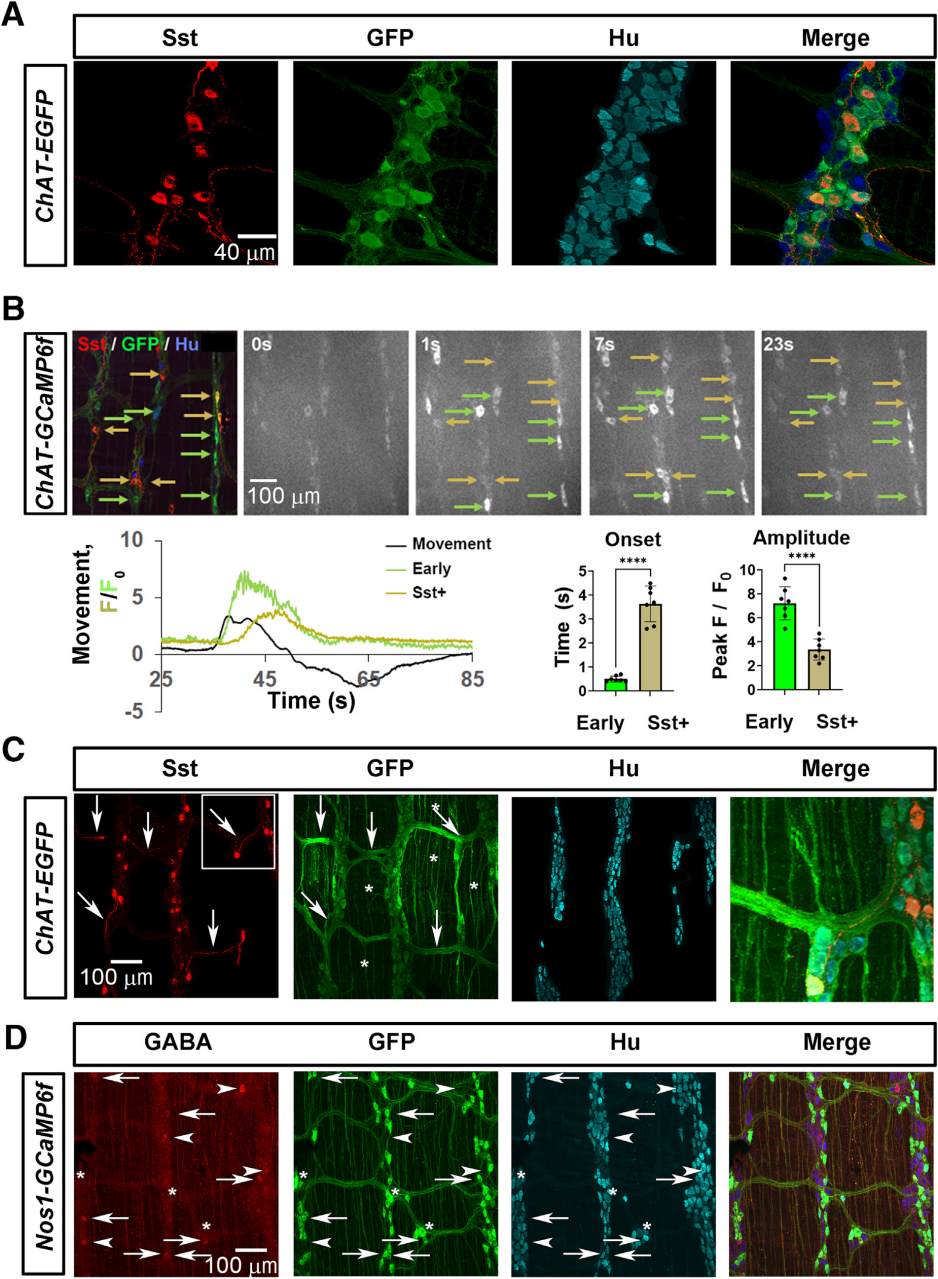
**Identification of molecular subclasses of cholinergic and nitrergic subtypes of myenteric neurons.** (*A*) Sst is found in cholinergic myenteric neurons of the middle colon. Antibodies against Sst, GFP, and Hu were used to stain Sst+, cholinergic, and all neurons of the middle colon from a *ChAT-eGFP* mouse. All Sst+ neurons were cholinergic (c = 67; n = 3 M, 3 F *ChAT-eGFP* mice). (*B*) *Post hoc* staining of Sst+ neurons in the middle colon of a *ChAT-GCaMP6f* mouse after imaging of cholinergic neurons during a contraction in response to distal mucosal stimulation. *Left image in upper panel* shows merged image of Sst, GFP, and Hu immunoreactivities of same ganglia imaged during contraction. *Right 4 images in upper panel* show fluorescence changes before and during the contractile movement, with *green arrows* denoting cholinergic neurons of the “early” subtype, and *gold arrows* indicating Sst+ neurons. One second after movement onset (1s), the “early” neurons become activated, whereas the Sst+ neurons only become activated later (7s). Averaged Ca^2+^ response of cholinergic neurons of the early subtype (*green*) compared with that of the Sst+ neurons (*gold*), plotted together with contractile movement track (*black*). Note the delayed onset and lower amplitude of the Sst+ neuronal response (c = 36 Sst+ and 36 early neurons; n = 3 M, 2 F). (*C*) Sst+ axons (*red*) travel through internodal strands (*arrows*), rather than parallel to the muscle (*asterisks*), as many other GFP+ cholinergic axons do (*green*). (*D*) *Post hoc* staining of GABA+ neurons the middle colon of a *Nos1-GCaMP6f* mouse after imaging of nitrergic neurons during a contraction in response to distal mucosal stimulation. Antibodies against GABA, GFP, and Hu were used to stain GABA+, nitrergic, and all neurons of the middle colon from a *Nos1-GCaMP6f* mouse. *Arrows* point to GABA+ neurons that are nitrergic (ie, GFP+), *arrowheads* point to GABA+ neurons that are not nitrergic (ie, GFP-negative), and *asterisks* indicate nitrergic long-on neurons. GABA+ neurons include nitrergic as well as non-nitrergic neurons, but not the long-on subpopulation of nitrergic neurons (56.4 ± 26% “off” nitrergic vs 43.6 ± 26% non-nitrergic; c = 22 Nos1+, 17 Nos1-negative; n = 3 M, 3 F).

We first demonstrated that Sst+ neurons of the myenteric plexus of the middle colon were exclusively cholinergic by co-labeling *ChAT-eGFP* mice with Sst and GFP antibodies (Figure 10*A*). Then, we labeled the middle colon of *ChAT-GCaMP6f* mice with these antibodies after imaging the responses of cholinergic neurons during neurogenic contractions evoked by stimulation of the distal mucosa. We observed that every cholinergic neuron immunolabeled *post-hoc* with Sst antibodies were of the middle subtype, with onsets occurring 3 to 4 seconds after contractile movement onset, in contrast to the early subtype, whose onsets were closer to 1 second after movement onset (Supplementary Video 10; Figure 10*B*). This homogeneity in functional response of Sst+ neurons in the middle colon during a neurogenic contraction was accompanied by a small range of somal area (265.9 ± 30.3 μm^2^, vs. 287.6 ± 31.7 μm^2^ of early cholinergic neurons, vs 179.4 ± 35 μm^2^ of transient cholinergic neurons; c = 95 Sst+ neurons, 42 early neurons, 39 transient neurons; n = 3 M, 3 F). The delay in response of Sst+ neurons after movement onset suggests that they are INs rather than deformation- or stretch-sensitive IPANs or motor neurons, whose onset would likely be just before or at the onset of the contractile movement. Evidence supporting this interpretation comes from the analysis of Sst+ axons, which travel exclusively through the internodal strands of the myenteric plexus of the middle colon, rather than out of the plexus toward the mucosa, or parallel to the circular or longitudinal muscle fibers (Figure 10*C*).

Next, we tested the hypothesis that the “long-on” nitrergic subtype reflected the GAD2+ transcriptomic subclass of enteric neuron,^38^^,^^39^ based on the onset of activity (ie, after contraction onset) and the large size of this subtype, which together suggest the possibility that these neurons are INs with long descending axons.^45^, ^46^, ^47^, ^48^ Nitrergic neurons of the middle colon of *Nos1-GCaMP6f* mice were immunostained *post-hoc* with antibodies against GABA, the product of both GAD1 (GAD67) and GAD2 (GAD65), as GAD2 antibody labeling was ineffective in our hands. Despite the caveat that GABA+ neurons likely include both GAD1+ and GAD2+ subpopulations, we failed to observe any overlap between GABA and “long on” nitrergic neurons (Figure 10*D*). Instead, roughly one-half of GABA+ neurons labeled “off” nitrergic neurons, and the other one-half labeled non-nitrergic neurons. GABA+ “off” nitrergic neurons were similar in size to GABA+ non-nitrergic neurons (somal area = 181.2 ± 30.8 vs 224.2 ± 71.4 μm^2^; c = 19 Nos1+, 11 Nos1-negative; n = 3 M, 3 F). GABA immunoreactivity in axons was observed in internodal strands as well as parallel to circular muscle fibers, consistent with a previous study.^49^ Together, these data demonstrate that specific subpopulations of enteric neurons defined by molecular transcriptomics exhibit distinct signatures of functional activity during neurogenic contractions of the intact colon.

## Conclusions

An understanding of the precise enteric neural circuits driving gastrointestinal motility is critical for the development of therapeutics designed to ameliorate various forms of dysmotility, which can occur on their own or as a part of a variety of inflammatory or neurodegenerative diseases.^50^^,^^51^ Although the basic neurochemical mediators of colonic motility have been established, many issues still remain. In particular, identification of the myenteric neural substrates underlying specific motility patterns has been plagued by methodological issues, most notably the presence of contractile movements that are required for the full expression of motility-associated neuronal activity, yet complicate measurement of it by tools such as intracellular recording or Ca^2+^ imaging. Although paralytic drugs have been used in some studies to address this problem and appear to retain some degree of neuronal activity in mouse colon,^18^ our results show that not only does this approach eliminate the movement itself, thus making a precise temporal correlation of neuronal activity patterns and the contractile movements they orchestrate impossible, but also dramatically reduces the activity of the neurons themselves, an effect recently also reported by Amedzrovi Agbesi et al.^34^ Therefore, in this study, we developed a preparation of whole colon that allowed for a degree of contractile-associated movement and thus the manifestation of robust neuronal activity patterns. Together with *post-hoc* motion subtraction techniques, we were able to measure these neuronal activity patterns and correlate them with the onset and offset of the contractile-associated movements, thus arriving at distinct neuronal substrates that underlie the contractions themselves as well as those associated with distinct stimuli that evoke them. Using this preparation and comparing the imaging results obtained to those collected from 3-channel tension recordings of colon, we were able to make several key observations.

First, in contrast to a previous neuronal Ca^2+^ imaging study in mouse colon, which found that all myenteric neurons exhibited synchronized activity at a frequency of 2 Hz during spont NgC,^18^ we observed distinct temporal patterns of activity within specific subpopulations of cholinergic and nitrergic neurons during these contractions. Some of these subtypes included neurons that were not active at all, including one-half of all nitrergic neurons, which respond robustly in between these contractions but abruptly shut off during them. We ascribe the differences between this and our study to the approach: whereas the previous study utilized Fluo4-loaded tissue, which labels all cell types, the current study used cell-specific GCaMP expression. As a result, transients appearing to derive from neurons in the previous study may have derived from surrounding CM or LM, which exhibits activity at 2 Hz^8^; indeed, we were unable to obtain valid Ca^2+^ transients from myenteric neurons or even ICCs in *CAGGS-GCaMP3* mice used in Figures 1 and 2, because the strength of the signal in the LM and CM contaminated the signal produced by these cell types.

Because both cholinergic and nitrergic neurons include multiple functional subtypes, it is tempting to speculate that some of the response subtypes reported in the current study correspond to these functional subtypes. For example, early and middle cholinergic subtypes likely include excitatory motor neurons (MNs), as these subtypes each exhibited the ∼2 Hz oscillation that matches the frequency of smooth muscle depolarization itself. Because LM activation preceded CM activation, it is possible that the early subtype reflects MNs that innervate this muscle. Direct evidence that a subpopulation of middle cholinergic subtypes are INs comes from the post-hoc labeling of them with Sst antibodies; because the axons of Sst+ neurons do not appear to innervate muscle or depart from the internodal strands in the middle colon, they are unlikely to represent MNs or IPANs. The nitrergic neurons that exhibit an increase of synchronized activity just before neurogenic contractions, then shut off during them, are likely to include at least CM-innervating if not also LM-innervating inhibitory MNs, because it has been shown that the activity of each of these subtypes increases just before and then is strongly reduced during a contractile event.^8^^,^^10^^,^^52^ By contrast, the nitrergic neuronal subtypes that exhibit activity just after contraction onset and persist during the contraction may represent long descending INs observed in guinea pig small intestine^45^^,^^46^ and human colon.^47^^,^^48^ These neurons likely contribute to the long-range coordination of spont NgC, perhaps by transferring the temporal information of a contraction in a proximal segment to a more distal segment. Indeed, the enhanced frequency of CMMCs observed in response to L-NNA suggest that central NO regulates the timing of propagation. However, because the only nitrergic neurons expressing GABA are the putative inhibitory MNs that cease activity just before the contraction, it appears unlikely that nitrergic neurons that turn on shortly after contractile onset are the GAD2+ subtype identified in transcriptomic studies.^38^^,^^39^

The neuronal substrates underlying a spont NgC appeared identical to those underlying an NgC elicited by a variety of stimuli, including mucosal stimulation, longitudinal stretch, or EFS of the proximal or distal colon, and PNS. These data suggest that the contractile event is driven by a common neuronal substrate, or final common output, that is itself activated by distinct stimuli and are corroborated by the 3-channel tension recordings, which show that each of these distinct stimuli produce a common pattern of anterogradely propagating contractions of similar amplitude, duration, and propagation speed. In this sense, the contractile substrate may represent a central pattern generator that is modified by distinct sensory inputs.^53^ The one exception to this finding was the variability of response to EFS of the proximal colon, which produced the same neuronal contractile substrate in the middle colon as each of the other stimuli in only some cases, whereas in other cases produced a slow accumulation of neuronal activity that produced a contraction that followed EFS termination. The reason for this is unclear but may depend on the difference between physiologic vs electrical activation of the proximal colon. Whereas stretch or mucosal deformation likely activates selective populations of myenteric neurons as a result of changes in the activity of the distal afferents of IPAN in mucosa and smooth muscle, EFS directly activates all myenteric neurons between the electrodes.

Three-channel tension recordings often showed an interesting feature: in response to each of the stimuli, similar to spontaneous CMMC, small, retrogradely propagating distal contractions were observed that preceded the larger, anterogradely propagating contractions that initiated in the proximal colon. Each of these occurred with faster onset in response to stimulation of distal than of proximal colon. These differences were reflected by distinct neuronal activity patterns in the middle colon that preceded the common contractile neuronal substrate. In response to all forms of stimulation of the proximal but not distal colon, a significant subpopulation of cholinergic, and nearly all nitrergic, neurons were active for the duration of the stimulus, before the ensuing contraction. These results are consistent with previous studies, one of which found a hyperpolarization that occurred in the middle region of the guinea pig distal colon during the length of time a distending stimulus was placed proximal to it, followed by a depolarization,^54^ and a second of which found both inhibitory and excitatory junction potentials in distal regions of colon during a distending stimulus placed at the proximal end.^55^ Collectively, these results suggest that distal stimulation produces a distal colonic pre-contraction that retrogradely propagates to the proximal colon and initiates the larger propagating contraction there, whereas proximal stimulation produces a pan-colonic relaxation that lasts the length of the stimulus together with the distal colonic pre-contraction, resulting in a slightly delayed anterogradely propagating contraction. A precedent for distal pre-contraction exists in the defecation motor program in *C. elegans*, wherein posterior body wall contraction precedes anterior body wall contraction, which then activates enteric neurons that excite enteric muscle contraction and propel fecal content.^56^ These findings suggest that in an intact colon stretched with pins (imaging preparation) or over a pellet-containing rod (tension recording preparation), the dominant motor pattern appears less similar to a traveling peristaltic reflex^57^ than to a pan-colonic anterogradely propagating contractile pattern that can be as efficiently triggered by descending as ascending stimuli.

Finally, we demonstrated that, although NO itself is dispensable for CMMC generation and propagation, other molecules within nitrergic neurons contribute to CMMC propagation. Some of these substances may include adenosine triphosphate (ATP), vasoactive intestinal peptide (VIP), pituitary adenylate cyclase-activating peptide (PACAP), galanin, and neuropeptide Y, which have been demonstrated in nitrergic neurons via immunohistochemical or transcriptomic approaches.^36^, ^37^, ^38^, ^39^, ^40^ Whether these factors affect propagation by an effect on Nos1-expressing inhibitory MNs or INs is unclear but can be addressed by future studies using specific genetic or pharmacologic approaches.

In conclusion, our imaging studies of the middle colon of *ChAT-GCaMP6f* and *Nos1-GCaMP6f* mice, coupled with tension recordings of whole colon, revealed the presence of a common neurogenic contractile substrate as well as stimuli-specific pre-contractile substrates that shed light on the mechanisms underlying some colonic motility patterns. The observations not only provide insight into the neuronal substrates underlying colonic motility patterns in normal conditions but offer a new tool to evaluate the activity of these substrates in pathologic conditions that exhibit disrupted motility.

## Methods

### Ethical Approval and Use of Mice

*ChAT-eGFP* mice (Jax #007902) were used to track cholinergic neurons in immunohistochemical experiments. *ChAT-Cre* (MMRRC #37336), and *Nos1-CreER*^*T2*^ mice (Jax #014541) were crossed to homozygous conditional tdTomato (tdTomato^flox/+^) mice (Jax #007909), GCaMP6f (GCaMP6f^flox/+^) mice (Jax #028865), or hm4Di (hM4Di^flox/+^) mice (Jax #026219) and maintained in the C57/Bl6 strain, producing *ChAT-Cre, GCaMP6f*^*Flox/+*^ or *ChAT-Cre, hM4Di*^*lox/+*^ mice, and *Nos1-CreER*^*T2*^*, tdTomato*^*Flox/+*^, *Nos1-CreER*^*T2*^*, GCaMP6f*^*Flox/+*^ or *Nos1-CreER*^*T2*^*, hM4Dif*^*Flox/+*^ mice. *ChAT-Cre, GCaMP6f*^*Flox/+*^ and *Nos1-CreER*^*T2*^*, GCaMP6f*^*Flox+x*^ mice were crossed again to GCaMP6f^flox /+^ mice to create *ChAT-Cre, GCaMP6f*^*Flox/flox*^ and *Nos1-CreER*^*T2*^*, GCaMP6f*^*Flox/flox*^ mice (hereafter termed *ChAT-GCaMP6f* or *Nos1-GCaMP6f* mice). *Kit-CreER*^*T2*^ mice (obtained from Dieter Saur; Technical University Munich)^58^ were also crossed to *GCaMP6f*^*Flox/+*^ mice to create *Kit-CreER*^*T2*^*, GCaMP6f*^*Flox/+*^ (*Kit-GCaMP6f*) mice, which were used to examine responses in ICC. *CAGGS-GCaMP3* transgenic mice, which express GCaMP3 under the control of the CMV-chick β-actin hybrid promoter^59^ that is activated by most cells in the mouse, were generated by S. Pfaff (Salk Institute) and published on before.^19^ *Nos1-tdTomato*, *Nos1-GCaMP6f*, *Nos1-hM4Di*, and *Kit-GCaMP6f* mice were treated with tamoxifen-containing food for 7 days at 5 to 6 weeks of age to induce Cre activity. M and F mice were sacrificed at a variety of ages between 8 and 16 weeks. Animal euthanasia, husbandry, and experiments were performed in accordance with the National Institutes of Health Guide for the Care and Use of Laboratory Animals, and the protocol was approved by the Institutional Animal Care and Use Committee at the University of Nevada, Reno.

### Drugs

Hex (100 mM stock dissolved in water), L-NNA (100 mM stock dissolved in 0.1 N HCl), and nifedipine (10 mM stock dissolved in ethanol) were purchased from Sigma-Aldrich. Clozapine-N-Oxide (2.5 mM stock dissolved in water) and TTX (1 mM stock in sodium citrate/citric acid) was purchased from Cayman. Tamoxifen-containing food was purchased from Envigo (TD.130855, 250 mg/kg diet).

### Preparation for imaging Colon

Adult mice of each sex were killed by cervical dislocation, in accordance with the requirements of the Animal Ethics Committee at the University of Nevada, Reno. A ventral midline incision was made, and the entire colon was removed and placed in a 6-cm dish lined with sylgard (Dow-Corning). The contents of the colon were gently flushed over the course of half an hour. Then, the middle of the intact colon (∼12 mm in length) was split and pinned flat, mucosa-side down. In our previous *ex vivo* imaging study of the muscle and myenteric ganglia of colons from *ChAT-GCaMP6f* and *Nos1-GCaMP6f* mice,^60^ we cut and pinned into a flat sheet the middle colon to a width of 7 to 8 mm, a level that: (1) approximates the circumference of a mouse pellet^61^^,^^62^; (2) maintains sufficient stretch, thus permitting the observation of periodic, large contractile movements that originate elsewhere and pass through the middle colon; (3) fails to evoke acute, stretch-induced contractile movements of the middle colon,^63^ therefore allowing the observation of tonic inhibition in between these periodic, large movements; and (4) prevents the full expression of the large contractile movement, thus permitting the tracking of Ca^2+^ responses in individual neurons. However, the movement that did occur at this level of stretch, as measured by optical displacement of an ROI that stayed within focus throughout the video, was shorter in duration than the increase of tension recorded from the outside of the middle of the intact whole colon by force transducers during a CMMC (19.3 ± 4.2 seconds; c = 20; n = 3 M, 3 F vs 41.6 ± 5.6 seconds; c = 14; n = 3 M, 5 F). This is consistent with the observation that sustained, suprathreshold circumferential stretch inhibits contractile activity in flat sheets of guinea pig ileum.^63^ Thus, we chose to place less stretch on the middle colon by pinning it to a width of 6 mm, to allow for more contractile movement to occur during a periodic contraction. Throughout this study, we imaged contractile movements and Ca^2+^ signals in this flat sheet preparation of middle colon during spontaneous as well as evoked neurogenic contractions (Figure 1*A*). The preparation was then perfused at 6 mL/min with Krebs-Ringer buffer (KRB; composition [mM]: NaCl: 118; KCl: 4.7; NaHCO_3_: 25; MgCl_2_: 1.2; NaH_2_PO_4_: 1.2; glucose: 11; CaCl_2_: 2.5; pH: 7.4; 21 °C) that was gassed with 95% O_2_, 5% CO_2_, and heated before the dish by a temperature controller (TC-344C; Warner Instruments) to 35 ^o^C to 37 ^o^C .

### Ca^2+^ Imaging and Movement Capture

Ca^2+^ imaging recordings (2-minute videos) were performed on a Nikon FN-1 upright microscope using 20× Fluor water-immersion lenses. Tissue was illuminated with a Spectra X light engine (Lumencor). Image sequences were captured using a Prime 95B Camera (Photometrics) and a Windows-based PC using Nikon NIS Elements 4.1. Image sequences were recorded at 20 frames per second and exported as 16-bit TIFF files into custom-written software (Volumetry G8d).^64^ An ROI was placed over a region that stayed within focus during the entire movie, and this was captured as a track and used to measure the displacement of the tissue. This displacement track was then used as a template to subtract the motion that occurred during the contraction so that the onset and duration of Ca^2+^ responses could be captured and compared with those of the movement. Background and ROI responses were calculated as spatial maps of the standard deviation (SD) of fluorescence (SD maps) from beginning to end of each video as previously described^19^ and were color-coded using a “Fire” color lookup table. Background-subtracted traces of fluorescence intensity were generated from movies, and the first value was normalized by first adding or subtracting 16-bit intensity units (iu_16_) to obtain a starting value of 1000 and then divided by 1000 to obtain a starting value (F_0_) of 1. Changes in fluorescence were occasionally reported as raw iu_16_ values but usually reported as value of fluorescence divided by initial fluorescence (F/F_o_).

### Spontaneous and Evoked Contractions of Colon

The colon was opened along a 10-mm length at either the oral or anal ends and pinned flat, serosa-side down, and the middle colon, prepared as stated above, was imaged. Two-minute videos were continuously recorded until spontaneous CMMC occurred. For mucosal stimulation, 30 seconds into the 2-minute video, 5 brush strokes were applied to either the proximal or distal mucosa over the course of 5 seconds. For longitudinal stretch stimulation, a small piece of plastic was attached via gluture (Leedstone) to the oral or anal end of the colon, and one end of a piece of silk string (6.0) was glued to the plastic, while the other end was looped around a 10-gm weight, which was placed on the stage outside the dish. Thirty seconds into the video, the weight was gently lowered for 5 seconds over the edge of the stage to stretch the colon longitudinally by an additional 10% of its length. For direct transmural EFS, 2 platinum wire electrodes separated by 3 to 5 mm were placed on top or underneath regions of the colon near the proximal or distal ends of the colon, and 10 seconds of 20 Hz stimulation (0.3 ms pulsewidth) was delivered 30 seconds into the video using an S88 square wave stimulator (Natus). For PNS, the entire tuft of tissue entering the sacral region of the colon was dissected^65^and placed into a suction electrode, and 10 s of 20 Hz stimulation (0.3 ms pulsewidth) was delivered 30 seconds into the video.

### Tension Recording of Split Middle Colon Preparation Used for Imaging

The middle colon was slit open as stated above and was pinned on one side only. A small piece of plastic with a hole in it was attached (via gluture) to the other side, and a single fiber of braided suture silk (6–0, Ethicon) was passed through a hole and attached to a force transducer and amplifier (Transbridge 4 M, WPI).

### Tension Recording of Proximal, Middle, and Distal Whole Colon: CMMC and Evoked Contractions

Whole colons were dissected and gently flushed as described above. Then, a 1.0-mm diameter fire-polished capillary tube, with an artificial pellet placed and glued around the middle of the tube, was gently pushed through the colon. This tube was longer than the colon, and these protruding regions of the tube were fixed to the bottom of a sylgard-lined organ bath with metal clips. Suture silk was attached via gluture to the proximal, middle, and distal colon, and on the other side was attached to 3 force transducers (TST125C; Biopac Systems). Resting tension was initially set to 8 mN, monitored using an MP100 interface, and recorded on a PC running Acqknowledge software 3.2.6 (Biopac Systems). Oxygenated KRB was perfused through the organ bath at a rate of 12 mL/min, and the chamber underlying the bath was heated to 35 ^o^C to 37 ^o^C. The frequency of spontaneous CMMC, as well as the amplitudes and durations of proximal or middle colonic contractions during a CMMC, as well as propagation times between the peak proximal to peak middle contractions, and peak middle to peak distal contractions, were measured. For evoked propagating contractions: (1) the mucosa of proximal or distal ends was exposed and stimulated with a brush; (2) the proximal or distal ends of the colon were stretched; (3) the transmural wire electrodes were placed atop the proximal or distal colon; or (4) a suction electrode was placed around the pelvic nerve. All of these manipulations were performed similar to the description of these stimulations of the preparations used for Ca^2+^ imaging.

### Immunohistochemistry

For quantification of Nos1 and ChAT myenteric neurons, *Nos1-tdTomato*, *ChAT-EGFP* mice were treated with tamoxifen food for 2 weeks. Six weeks later, mice were sacrificed. *ChAT-GCaMP6f* mice were also sacrificed at 12 to 14 weeks of age. The mucosa and submucosa were removed with forceps from pinned, split whole colons, fixed for 2 hours in 4% paraformaldehyde, and incubated overnight at 4 ^o^C with antibody solution in phosphate buffered saline (PBS) containing 1% triton-X (1% PBST) and 10% fetal bovine serum (FBS) (ThermoFisher). Antibodies against Nos1 (rabbit, ThermoFisher), GFP (goat, Rockland Immunochemicals), somatostatin (rabbit, ABclonal), GABA (rabbit, MilliporeSigma), GAD65 (rabbit, ThermoFisher), or Hu/Hu C/D (human ANNA-1, kindly provided by Dr Vanda Lennon, Mayo Clinic) were diluted 1/1000 in 10% FBS/PBST (except GAD65, which was tried at a range of concentrations from 1/100 to 1/2000). Fluorescently conjugated secondary whole antibodies or Fab fragments (ThermoFisher or Jackson Immunoresearch) were added to rinsed whole mounts at 1/400 in 10% FBS/PBST overnight at 4 ^o^C. Tissues were rinsed, mounted with the longitudinal muscle nearest to the coverslip, and confocally imaged with Fluoview software controlling an Olympus FV-1000 laser-scanning fluorescence microscope (Olympus).

### Statistics

Results are shown as the mean ± SD. n represents the number of mice and c the number of samples (ie, cells, contractions). The minimum total number of animals chosen for any given experiment (n = 4) was determined by power analysis in G∗power 3.010 (alpha = .05; power = 80%), with an anticipated large effect size, based on our previous studies of similar contractile and Ca^2+^ activity phenomena).^60^ In several cases, we tested for sex differences and reported the data in the figure and legend (eg, contractile features during CMMC in Figure 4*A*, Nos1 Ca^2+^ response subtypes during a spont NgC in Figure 8*B*, or did not report the data (eg, ChAT Ca^2+^ response subtypes during a spont NgC in Figure 4*B*). In these cases, a sample size of at least 4 M and 4 F were evaluated. However, because there were no sex differences in these contractile and cholinergic or nitrergic neuronal activity responses during spont NgC, we pooled the results from M and F for the rest of the study. When samples were compared between different animals, unpaired Student *t*-tests or 1-way analyses of variance (ANOVA) were used (eg, frequency amplitudes, durations, propagation times of CMMC, or evoked contractions). When the same samples were being compared, paired Student *t*-tests or ANOVAs were used (eg, neurons responding in a spontaneous vs evoked neurogenic contraction, contractions pre- and post-drug). Most *t*-tests were run with 2 tails, except those for which the effect was only examined in 1-way based on expectation. Bonferroni’s correction was used when comparing certain samples (eg, pre-contraction onset after EFS vs PNS). After ANOVAs, comparisons between individual values were assessed by Dunnet’s multiple comparison test. Differences in value were recognized as significant at *P* > .05(∗). Cumulative temporal correlation distributions of Ca^2+^ responses during tonic inhibitory periods were evaluated with the Kolmogorov-Smirnov test. All statistical comparisons are available in uploaded raw data files. Statistical tests and graphs were generated by Microsoft Excel or Graphpad Prism. All authors had access to the study data and had reviewed and approved the final manuscript.

## Supplementary Material

Supplementary Video 1 A spontaneous neurogenic contraction (spont NgC) and pan-cellular Ca^2+^ response that occurs in *CAGGS-GCaMP3* mice. Note the slight rightward movement toward distal colon, coupled with longitudinal muscle transients, that precede circular muscle and neuronal transients. Note also the return to phasic contractile movements and circular muscle transients after the neurogenic contraction.

Supplementary Video 2 A spont NgC and cholinergic Ca^2+^ response that occurs in *ChAT-GCaMP6f* mice. This video corresponds to the spatial map of fluorescence of Figure 4. Note the heterogeneity of response, with some active during the initial rightward movement, followed by others at the onset of the leftward movement, followed by others slightly later, during the peak contractile period of limited movement.

Supplementary Video 3 A second spont NgC and cholinergic Ca^2+^ response that occurs in *ChAT-GCaMP6f* mice.

Supplementary Video 4 A neurogenic contraction and cholinergic Ca^2+^ response, evoked by brush stimulation of the proximal mucosa, that occurs in the same neurons as in Supplementary Video 3. Note that the same subtypes that are active during a spont NgC are also active in response to a contraction triggered by this stimulation. Note, as well, the response of some cholinergic neurons during the brush stimulation itself, 25% into the video, and before the subsequent contraction and contractile-associated responses.

Supplementary Video 5 A neurogenic contraction and cholinergic Ca^2+^ response, evoked by brush stimulation of the distal mucosa, that occurs in the same neurons as in Supplementary Videos 3 and 4. Note that the same subtypes active during a spont NgC are active in response to a contraction triggered by this stimulation. Note, as well, the absence of the brush stimulation-associated response.

Supplementary Video 6 A spont NgC and nitrergic Ca^2+^ response that occurs in *Nos1-GCaMP6f* mice. Note the increasing apparent synchrony of nitrergic neurons, during the tonic inhibitory period, just before the contraction. Note as well the turning off of many nitrergic neurons during the contraction, as well as the turning on of others just after contraction onset. This video corresponds to the spatial map of fluorescence of Figure 8.

Supplementary Video 7 The same video as Supplementary Video 6, except after motion stabilization. Note the turning off of cells 1 to 5 from Figure 8, as well as the turning on of cells 6 to 9. The response by cell 9 lasts the longest and reflects a nitrergic subtype that has the largest somal area (see text).

Supplementary Video 8 A neurogenic contraction and nitrergic Ca^2+^ response, evoked by brush stimulation of the proximal mucosa. Note the response of most nitrergic neurons during the brush stimulation itself, 25% into the video, and before the subsequent contraction and contractile-associated responses.

Supplementary Video 9 A neurogenic contraction and nitrergic Ca^2+^ response, evoked by brush stimulation of the distal mucosa, that occurs in the same neurons as those in Supplementary Video 8. Note the absence of the brush stimulation-associated response.

Supplementary Video 10 A neurogenic contraction and cholinergic Ca^2+^ response, evoked by stimulation of the distal colon, after motion stabilization. Images and averaged transients from this video and others like it are shown in Figure 10*B*; *green box* shows 2 early-subtype cholinergic neurons and *brown box* 2 Sst+ middle-subtype cholinergic neurons that are also identified in Figure 10*B*.
